# Draft genome sequences of strains CBS6241 and CBS6242 of the basidiomycetous yeast *Filobasidium floriforme*

**DOI:** 10.1093/g3journal/jkab398

**Published:** 2021-11-15

**Authors:** Marco Alexandre Guerreiro, Steven Ahrendt, Jasmyn Pangilinan, Cindy Chen, Mi Yan, Anna Lipzen, Kerrie Barry, Igor V Grigoriev, Dominik Begerow, Minou Nowrousian

**Affiliations:** 1 Department of Evolution of Plants and Fungi, Ruhr-Universität Bochum, Bochum 44801, Germany; 2 U.S. Department of Energy Joint Genome Institute, Lawrence Berkeley National Laboratory, Berkeley, CA 94720, USA; 3 Department of Plant and Microbial Biology, University of California Berkeley, Berkeley, CA 94720, USA; 4 Department of Molecular and Cellular Botany, Ruhr-Universität Bochum, Bochum 44801, Germany

**Keywords:** *Filobasidium floriforme*, mating-type locus, basidiomycete, Filobasidiales, CAZymes

## Abstract

The Tremellomycetes are a species-rich group within the basidiomycete fungi; however, most analyses of this group to date have focused on pathogenic Cryptococcus species within the order Tremellales. Recent genome-assisted studies of other Tremellomycetes have identified interesting features with respect to biotechnological applications as well as the evolution of genes involved in mating and sexual development. Here, we report genome sequences of two strains of *Filobasidium floriforme*, a species from the order Filobasidiales, which branches basally to the Tremellales, Trichosporonales, and Holtermanniales. The assembled genomes of strains CBS6241 and CBS6242 are 27.4 Mb and 26.4 Mb in size, respectively, with 8314 and 7695 predicted protein-coding genes. Overall sequence identity at nucleic acid level between the strains is 97%. Among the predicted genes are pheromone precursor and pheromone receptor genes as well as two genes encoding homedomain (HD) transcription factors, which are predicted to be part of the mating type (*MAT*) locus. Sequence analysis indicates that CBS6241 and CBS6242 carry different alleles for both the pheromone/receptor genes as well as the HD transcription factors. Orthology inference identified 1482 orthogroups exclusively found in *F. floriforme*, some of which were involved in carbohydrate transport and metabolism. Subsequent CAZyme repertoire characterization identified 267 and 247 enzymes for CBS6241 and CBS6242, respectively, the second highest number of CAZymes among the analyzed Tremellomycete species. In addition, *F. floriforme* contains five CAZymes absent in other species and several plant-cell-wall degrading CAZymes with the highest copy number in Tremellomycota, indicating the biotechnological potential of this species.

## Introduction

The basidiomycete group of Tremellomycetes is a species-rich group comprising both filamentous and yeast-like fungi (Liu *et al.* 2015a, 2015b; Spatafora *et al.* 2017). In-depth studies in this group have mostly focused on pathogenic Cryptococcus species (order Tremellales) (Sun *et al.* 2019a; Bahn *et al.* 2020). However, facilitated by increased genome-sequencing capacities in recent years, additional species within the Tremellomycetes have been investigated, often for their biotechnological potential or to study the evolution of sexual development in this group (Sharma *et al.* 2015; Bellora *et al.* 2016; Barredo *et al.* 2017; Bracharz *et al.* 2017; Coelho *et al.* 2017; Sun *et al.* 2019b; Aliyu *et al.* 2020).

Mating and sexual development in basidiomycetes is regulated by mating type (*MAT*) genes encoding pheromone precursors, pheromone receptors, and homeodomain (HD) transcription factors. The ancestral state in basidiomycetes is thought to be tetrapolar, with two nonlinked genetic loci containing pheromone and pheromone receptor genes (*P/R* locus) and HD transcription factor genes (*HD* locus), respectively (Kües *et al.* 2011; Coelho *et al.* 2017). However, studies of pathogenic Cryptococcus species revealed a single, large *MAT* locus predicted to have arisen from genomic transitions leading to fusion of the formerly unlinked *P/R* and *HD* loci (Lengeler *et al.* 2002; Fraser *et al.* 2004), whereas in all other species of the order Tremellales that were analyzed so far, the ancient tetrapolar arrangement of unlinked *P/R* and *HD* loci is found (Sun *et al.* 2019a). In contrast, in the Trichosporonales, the sister order to the Tremellales (Liu *et al.* 2015b), a recent analysis of *MAT* loci revealed that in all analyzed species, *P/R* and *HD* loci are physically linked in a single *MAT* locus (Sun *et al.* 2019b).

For the Tremellomycete order Filobasidiales, which branches basally to the Tremellales, Trichosporonales, and Holtermanniales (Liu *et al.* 2015b), genomes have been published for the genera Naganishia and Solicoccozyma, but none were analyzed with respect to the mating type (Close *et al.* 2016; Vajpeyi and Chandran 2016; Yong *et al.* 2016; Bijlani *et al.* 2020; Han *et al.* 2020; Nizovoy *et al.* 2021). Here, we present the first draft genome sequences including an analysis of *MAT* genes for two strains of the genus Filobasidium, *Filobasidium floriforme* strains CBS6241 and CBS6242.

Carbohydrate-active enzymes (CAZymes) are essential for fungi as heterotrophic organisms. These enzymes are responsible for the biosynthesis, modification, binding, and breakdown of carbohydrates and glycoconjugates (Cantarel *et al.* 2009). Different fungal species harbor different sets of CAZymes to meet their ecological needs as saprobes, symbionts, endophytes, parasites, or pathogens (Rytioja *et al.* 2014; Kameshwar and Qin 2018). CAZyme content and diversity is therefore suggested to reflect niche adaptation. CAZymes are widely applied in various biotechnological processes and industries, such as in food, wine, paper, pulp, textile, detergents, biofuels, biorefinery, and bioremediation (Mäkelä *et al.* 2014). Since Tremellomycetes are known for their ability to colonize and inhabit a vast diversity of substrates, characterizing their set of CAZymes provides a great opportunity to identify and characterize species with biotechnological potential.

## Materials and methods

### Strains and culture conditions

Strains CBS6241 and CBS6242 were obtained from the Westerdijk Fungal Biodiversity Institute (Utrecht, The Netherlands). Strains were kept on YPD agar medium at 25°C.

### DNA extraction and sequencing

For DNA extraction, strains were grown as pre-cultures for 4 days at 25°C on YPD agar medium. From single colonies, 30 ml liquid YPD medium was inoculated and cultures were incubated at 25°C and 100 rpm on a shaker for 24 h (OD_600_ > 1). DNA was extracted as described previously (Kourist *et al.* 2015).

The genome of strain CBS6241 was sequenced using Pacific Biosciences Sequel sequencing platform. One microgram of genomic DNA was sheared to 10 kb using Covaris g-TUBE. The sheared DNA was treated with DNA damage repair mix followed by end repair and ligation of blunt adapters using SMRTbell Template Prep Kit 1.0 (Pacific Biosciences). The library was purified with AMPure PB beads. PacBio Sequencing primer was then annealed to the SMRTbell template library and sequencing polymerase was bound to them using Sequel Binding kit 3.0. The prepared SMRTbell template libraries were then sequenced on a Pacific Biosystem’s Sequel sequencer using v3 sequencing primer, 1M v3 SMRT cells, and Version 3.0 sequencing chemistry with 1 × 360 and 1 × 600 sequencing movie run times. A total of 3,594,174 reads were obtained with an average length of 3.8 kb and an N50 of 5.7 kb.

Library preparation and Illumina sequencing of strain CBS6242 was performed by Eurofins (Konstanz, Germany). Paired-end reads of 151 nt were sequenced from a library with an average insert size of 330 nt.

### RNA extraction, sequencing, and transcriptome assembly

For RNA extraction, CBS6241 was grown as pre-culture for 4 days at 25°C on YPD agar medium. From single colonies, 30 ml of three different liquid media were inoculated (V8: 50 ml/l vegetable juice, pH 5.2; V8-YPD1: 50 ml/l vegetable juice, 10 g/l tryptone, 5 g/l yeast extract, 10 g/l glucose, pH 5.2; V8-YPD2: 25 ml/l vegetable juice, 20 g/l tryptone, 5 g/l yeast extract, 10 g/l glucose, pH 5.2). Cultures were incubated at 25°C and 100 rpm on a shaker for 24 h. RNA was extracted as described previously (Kourist *et al.* 2015).

The transcriptome of strain CBS6241 was sequenced using Illumina 2 × 150 paired-end reads. Stranded cDNA library was generated using the Illumina Truseq Stranded RNA LT kit. mRNA was purified from 1 µg of total RNA using magnetic beads containing poly-T oligonucleotides. mRNA was fragmented and reversed transcribed using random hexamers and SSII (Invitrogen) followed by second strand synthesis. The fragmented cDNA was treated with end-pair, A-tailing, adapter ligation, and eight cycles of PCR. The prepared library was quantified using KAPA Biosystems’ next-generation sequencing library qPCR kit and run on a Roche LightCycler 480 real-time PCR instrument. Sequencing of the library was performed on the Illumina NovaSeq sequencer using NovaSeq XP V1 reagent kits, S4 flowcell, following a 2 × 150 dependent indexed run recipe. Raw reads were filtered and trimmed. Using BBDuk (https://sourceforge.net/projects/bbmap/, Accessed: 2021 November 19), raw reads were evaluated for artifact sequence by kmer matching (kmer = 25), allowing 1 mismatch and detected artifact was trimmed from the 3′ end of the reads. RNA spike-in reads, PhiX reads and reads containing any Ns were removed. Quality trimming was performed using the phred trimming method set at Q6. Finally, following trimming, reads under the length threshold were removed (minimum length 25 bases or 1/3 of the original read length—whichever is longer). Filtered reads were assembled into consensus sequences using Trinity (v2.3.2) (Grabherr *et al.* 2011), run with the –normalize_reads (In-silico normalization routine) and –jaccard_clip (Minimizing fusion transcripts derived from gene dense genomes) options. The transcriptome data were used for annotation.

### Genome assembly and annotation

Filtered Pacific Biosciences subread data for strain CBS6241 was filtered for artifacts and assembled with Falcon version pb-assembly = 0.0.2|falcon-kit = 1.2.3|pypeflow = 2.1.0 (https://github.com/PacificBiosciences/FALCON, Accessed: 2021 November 19) and polished with Arrow version SMRTLink v7.0.1.66975 (https://www.pacb.com/support/software-downloads, Accessed: 2021 November 19), improved with finisherSC version 2.1 (Lam *et al.* 2015), and polished with Arrow version SMRTLINK v7.0.1.66975 (https://www.pacb.com/support/software-downloads, Accessed: 2021 November 19). The genome was annotated using the JGI Annotation pipeline (Grigoriev *et al.* 2014).

For strain CBS6242, Illumina reads were quality-trimmed with Trimmomatic v0.36 (Bolger *et al.* 2014) and assembled with SPAdes v3.14.0 (Prjibelski *et al.* 2020). Contigs >1 kb were kept for downstream analyses. Contigs were error-corrected with Pilon v1.22 (Walker *et al.* 2014) based on the Illumina reads mapped to the assembly with Bowtie2 v2.2.6 (Langmead and Salzberg 2012). For CBS6242, genes were predicted with Maker v2.31.8 (Cantarel *et al.* 2008) based on the predicted genes of CBS6241. Pheromone genes in both strains were identified with a custom-made Perl script (Supplementary Text S1) to search for the consensus sequence M-X(15-60)-CAAX-Stop, with X representing any amino acid and A representing the amino acids valine, leucine, isoleucine, methionine, threonine or serine. Putative telomeric repeats (sequence TTAGGGG occurring consecutively at least three times) were identified with a custom-made Perl script (Supplementary Text S2).

### Phylogenetic analysis and functional annotation

Published genome assembly data of tremellomycetous species were collected from NCBI and JGI databases (Supplementary Table S1). The respective proteomes were predicted by Augustus version 3.3.3 (Stanke *et al.* 2008), with *Cryptococcus neoformans* as reference organism. Orthologous protein sequences were identified with OrthoFinder v2.5.2 (Emms and Kelly 2019). Single-copy orthologous sequences present in all species were individually aligned with MAFFT v7.273 (Katoh and Standley 2013) and concatenated. The maximum likelihood phylogenetic tree was calculated based on a single alignment of 142 single-copy orthologous genes by RAxML v8.2.12 (Stamatakis 2014) using 500 bootstrap replications, the PROTGAMMAWAG model, 123 as seed number for the parsimony inferences and a random seed of 321.

The strains *Cryptococcus deneoformans* JEC21 (Loftus *et al.* 2005), *Cutaneotrichosporon oleaginosum* IBC0246 (Kourist *et al.* 2015) and *Cystofilobasidium capitatum* CBS7420 (David-Palma *et al.* 2020) were selected for further analyses as representatives of Tremellales, Trichosporonales and Cystofilobasidiales, respectively (one species per order). Orthology comparisons were calculated with *ComplexUpset* (v1.3.1) package for R v4.1.0 and “exclusive intersection” mode.

Genes comprised in orthogroups exclusively found in *F. floriforme* were extracted and functionally annotated with PFAM v34.0 online database (Mistry *et al.* 2021) and eggNOG-mapper v2.1.0-1 (Huerta-Cepas *et al.* 2019; Cantalapiedra *et al.* 2021).

### Analysis of the *MAT* regions

Homologs to *MAT* genes were identified with BLAST analyses (Altschul *et al.* 1997) using MAT proteins from *Cryptococcus neoformans* (Lengeler *et al.* 2002) as queries. Phylogenetic trees of Ste3 proteins from *F. floriforme* strains and from published sequences of Trichosporonales and Tremellales species (Lengeler *et al.* 2000, 2002; Kourist *et al.* 2015; Takashima *et al.* 2015, 2018; Cho *et al.* 2016; Sriswasdi *et al.* 2016; Sun *et al.* 2019b) were generated with PAUP version 4.0b10 for Windows (D.L. Swofford, distributed by Sinauer Associates, copyright 2001 Smithsonian Institution) for Neighbor joining analyses or with MrBayes (Huelsenbeck and Ronquist 2001; Ronquist and Huelsenbeck 2003) based on multiple alignments generated with CLUSTALX (Thompson *et al.* 1997). Comparisons of genomic regions at nucleic acid level were performed with nucmer from the MUMmer package (Kurtz *et al.* 2004).

### Prediction of CAZymes

Proteomes of published datasets in Filobasidiales and of representative members of Tremellales, Trichosporonales and Cystofilobasidiales were scanned against dbCAN2 v9.0 database (Zhang *et al.* 2018) using HMMER v3.3.2 (Eddy 2011). Matches with an e-value lower than 1e-15 and a coverage higher than 0.35 were used for further analyses.

## Results and discussion

### Genome assembly and assessment

The genome of the *F. floriforme* strain CBS6241 was sequenced as part of the 1000 Fungal Genomes project (http://1000.fungalgenomes.org) (Grigoriev *et al.* 2011, 2014) using PacBio sequencing, while strain CBS6242 was sequenced with Illumina sequencing. With assembly sizes of 26–27 Mb and 7695 and 8314 predicted genes (Table 1), the genomes of the two *F. floriforme* strains are in the same range as the previously sequenced 24.8 Mb genomes of two strains of the Filobasidiales species *Naganishia albida*, for which 7375 and 8637 genes were predicted (Vajpeyi and Chandran 2016; Yong *et al.* 2016).

**Table 1:** Genome assembly statistics for CBS6241 and CBS6242

	CBS6241	CBS6242
Assembly size (Mb)	27.5	26.4
No. of scaffolds	42	700
N50 (kb)	9388	120
GC content (%)	52.9	52.9
Predicted genes	8314	7695
completeness (%)*a*	94.9	92.6
Coding regions (%)	46.0	44.3

a Completeness was analyzed with BUSCO v5.2.1 (Manni *et al.* 2021).

Searches for putative telomeric repeats identified seven contigs in CBS6241 with putative telomeric repeats at both ends, and 23 contigs with putative telomeric repeats at one end (Supplementary Table S2). This suggests that the genome of CBS6241 consists of (at least) 19 chromosomes. In the Illumina-sequenced genome of CBS6242, no putative telomeric repeats were identified, as is to be expected in a genome assembled from short reads.

### Phylogenetic analysis

**Figure 1:**
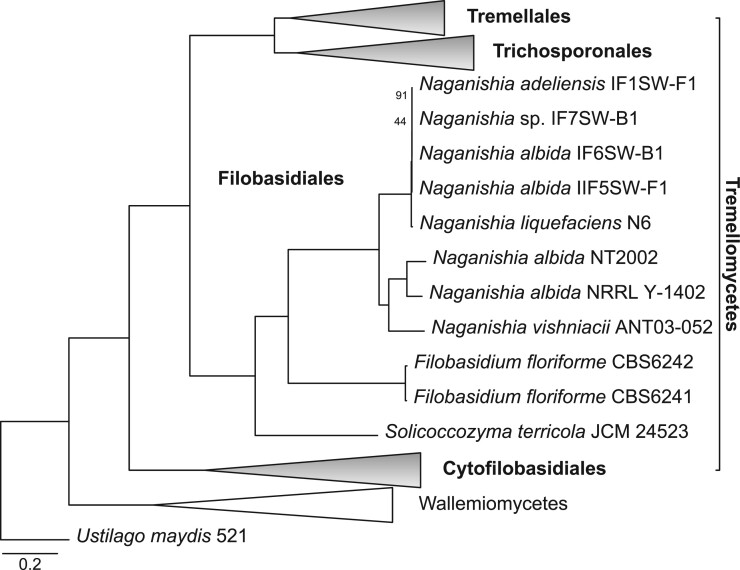
Phylogenetic analysis of Tremellomycetes. A maximum likelihood analysis of 142 single-copy orthologous protein sequences was performed with 500 bootstrap replicates using *Ustilago maydis* as an outgroup. All depicted nodes showed 100% bootstrap support except when noted. Accession numbers for the genome assembly data is provided in Supplementary Table S1. Groups outside of the Filobasidiales are collapsed, for the full phylogeny, see Supplementary Figure S1. The scale bar gives substitutions per site.

The maximum likelihood analysis of the 142 single-copy orthologous protein sequences produced a highly resolved phylogeny of Tremellomycota, in which most clades were supported by bootstrap values of 100% (Figure 1, Supplementary Figure S1). The Filobasidiales order is monophyletic and basal to the orders Tremellales and Trichosporonales. Cystofilobasidiales is the most basal order in Tremellomycota. The calculated phylogeny is consistent with previous reconstructions (Liu *et al.* 2015a, 2015b). Currently, classifications suggest that the Filobasidium genus is likely monophyletic and closely related to Naganishia and Solicoccozyma genera (Kwon-Chung 2011; Liu *et al.* 2015a), supporting our results. Due to their placement in the phylogeny (Figure 1, Supplementary Figure S1), a taxonomic revision of available Naganishia genomes might be advisable for future studies.

### Orthologous genes and functional assignment

**Figure 2:**
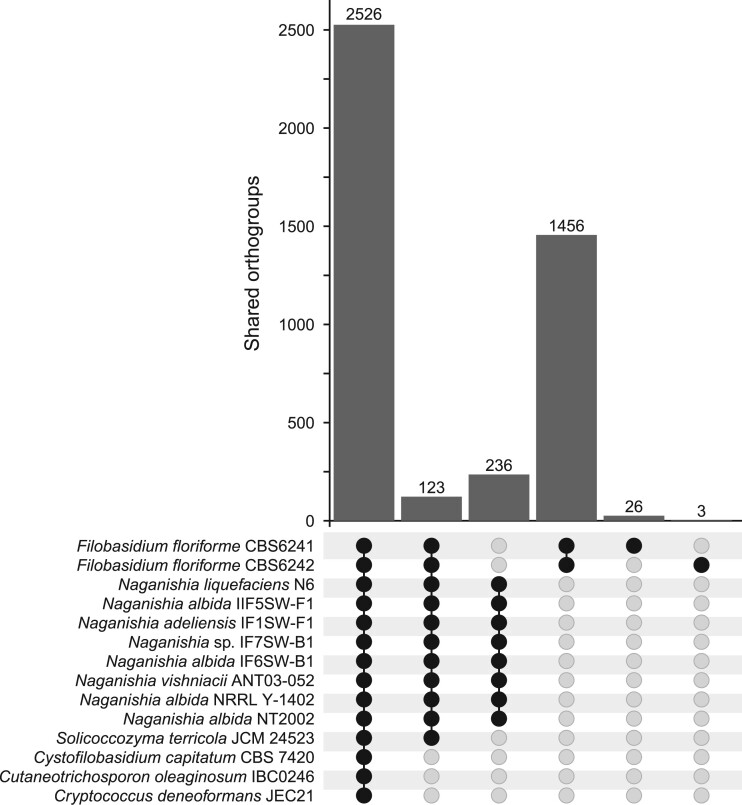
Orthogroup analysis to identify shared orthogroups between *F. floriforme* strains and other Tremellomycetes. Black dots indicate species/strains that share the indicated number of orthogroups. Indicated orthogroups are exclusively found in the indicated set of species [shared orthogroups were compared among orders (Tremellales, Trichosporonales, Filobasidiales, and Cystofilobasidiales) and within the Naganisha genus].

Orthology inference analysis revealed that 2526 orthogroups were shared among all the selected Tremellomycetes species (Figure 2). Furthermore, 123 were exclusively found among members of Filobasidiales, while 236 were exclusive to the Naganishia genus. A total of 1485 unique orthogroups were exclusively found in the two strains of *F. floriforme*, from which 83 orthogroups (comprising 224 orthologous sequences) were successfully functionally assigned by both PFAM and eggNOG databases (Supplementary Table S3). The most represented eggNOG functional categories included unknown function (31 orthogroups), carbohydrate transport and metabolism (13), post-translational modification, protein turnover, and chaperones (10), and replication, recombination, and repair (8). Functional description resulted, among others, in glycosyl hydrolases, dehydrogenases, kinases and proteases, that were specific to *F. floriforme*. These unique features might indicate a unique evolutionary adaptation to the environment (Rytioja *et al.* 2014; Nizovoy *et al.* 2021). Both strains comprised similar copy numbers, with a few exceptions in some domains, which could be explained by the different assembly quality of both genomes (Table 1). Orthology inference analysis and functional assignment suggest that *F. floriforme* might comprise unique genomic traits that might be valuable for further functional studies (Nizovoy *et al.* 2021).

### Analysis of *MAT* genes

Putative pheromone receptor genes and *HD* genes were identified by BLAST searches with the corresponding *C. neoformans* genes, whereas putative pheromone precursor genes were identified through searches for a consensus sequence with a custom-made Perl script. In each of the *F. floriforme* strains, one *STE3* pheromone receptor gene, one pheromone precursor gene, and two *HD* transcription factor genes (*SXI1* and *SXI2*) were identified (Supplementary Figures S2 and S3). The pheromone receptor gene and pheromone precursor gene of CBS6241 are located in a 21 kb region on contig 14, whereas the *HD* genes are located in a 4 kb region on contig 3. Both contigs have telomeric repeats at both ends (Supplementary Table S2), making it likely that they represent different chromosomes. This suggests that the *P/R* and *HD* loci of CBS6241 are genetically unlinked and that therefore the mating type configuration of *F. floriforme* might be tetrapolar.

The genomic regions containing the *HD* genes are syntenic in CBS6241 and CBS6242 (Supplementary Figure S4). The predicted pheromone precursor gene of CBS6242 is located toward one end of contig 18, and the region is syntenic to the corresponding region in CBS6241 (Supplementary Figure S4). However, the pheromone receptor genes *STE3* is located on a separate, short contig that shows two inversions compared to CBS6241 (Supplementary Figure S4). Furthermore, a repeat-rich region of 15 kb that is present close to *STE3* in CBS6241 was not assembled in CBS6242, probably due to the short read-based assembly. Repeat expansions and sequence divergence have been observed previously in the *MAT* regions of other basidiomycetes and are often associated with sex- or mating type-determining regions with reduced recombination (Lengeler *et al.* 2002; Loftus *et al.* 2005; Branco *et al.* 2017, 2018; Coelho *et al.* 2017).

**Figure 3:**
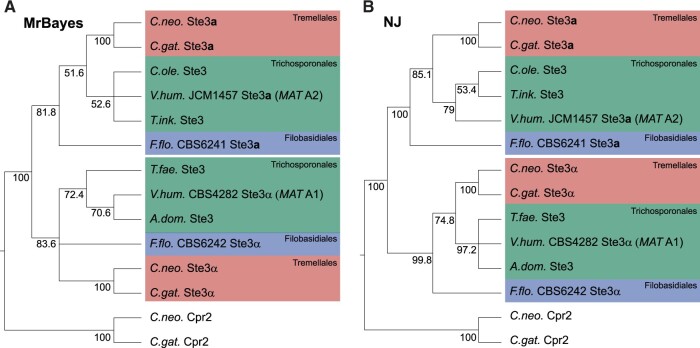
Phylogenetic analysis of Ste3 proteins from several Tremellomycetes. Analysis was done by MrBayes (A) or Neighbor joining (NJ, in B). Bayesian probabilities (A) or bootstrap percentages for 1000 bootstrap replications (B) are given at the branches. The pheromone-receptor-like Cpr2 proteins from *C. neoformans* and *C. gattii* were used as outgroups. The phylogenetic trees show a deep trans-species polymorphism for the Ste3 proteins within the Tremellomycetes that not only includes the sister orders Tremellales and Trichosporonales, but also includes the early-branching Filobasidiales represented by the two *F. floriforme* strains. Species abbreviations and accession numbers or locus tag numbers: *A. dom, Apiotrichum domesticum* (T. domesticum_002_745, genome accession BCFW01000000); *C. neo, Cryptococcus neoformans* (Ste3a: AAN75624.1, Ste3α: XP_012049557.1, Cpr2: XP_012047561.1); *C. gat, Cryptococcus gattii* (Ste3a: AEG78597.1, Ste3α: XP_003196044.1, Cpr2: XP_003191200.1); *C. ole, Cutaneotrichosporon oleaginosum* (XP_018276494.1); *F. flo, Filobasidium floriforme* (CBS6241: gene_1555, CBS6242: CBS6242_07693 = FFLO_06159), *T. fae, Trichosporon faecale* (T. faecale_002_949, genome accession JXYK01000000), *T. ink. Trichosporon inkin* (T. inkin_003_120, genome accession JXYM01000000); *V. hum, Vanrija humicola* (Ste3a: JCM1475_001_295, genome accession BCJF01000000, Ste3α: TXT13458.1).

The *STE3* gene of CBS6241 belongs to the *MAT***a** group of *STE3* pheromone receptor genes, whereas the *STE3* gene of CBS6242 belongs to the *MAT*α group of *STE3* genes (Figure 3, Supplementary Figure S2). A phylogenetic analysis of *STE3* alleles from the two *F. floriforme* strains as well as *STE3* genes from Trichosporonales and Tremellales showed trans-species polymorphism already observed for Trichosporonales and Tremellales (Metin *et al.* 2010; Findley *et al.* 2012; Sun *et al.* 2019b), indicating that the trans-species polymorphism of *STE3* alleles was present already in the last common ancestor of the Filobasidiales and its sister orders (Figure 3).

**Figure 4:**
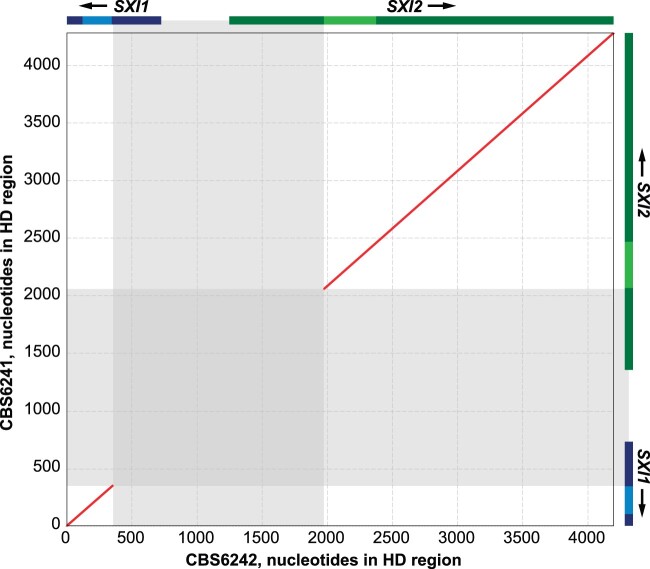
Dot plot of HD transcription factor gene regions of *F. floriforme* strains CBS6241 and CBS6242. The comparison was performed with nucmer from the MUMmer package (Kurtz *et al.* 2004). Nucleic acid sequence identity in the first aligned region (nt 1–356) is 96.4%, in the second aligned region (nt ∼2000–4200) it is 84.0%. No sequence similarity was detected in the region shaded in gray, which comprises the intergenic region and the N-terminal regions of *SXI1* and *SXI2*. The regions encoding the conserved homeodomains in Sxi1 and Sxi2 are shown in light blue and light green, respectively. Gene regions outside of the conserved homeodomain-encoding regions are given in dark blue and dark green for *SXI1* and *SXI2*, respectively. The sequences used for comparison comprise the following genomic regions in the CBS6241 and CBS6242 assemblies, respectively: CBS6241 contig14 nt 669,525–673,807, CBS6242 contig45 nt 55,021–59,216.

Both strains carry two *HD* genes (*SXI1* and *SXI2*) next to each other but divergently transcribed, which is the typical genomic arrangement for basidiomycete *HD* genes (Coelho *et al.* 2017). Sequence comparison of the *HD* region of CBS6241 and CBS6242 showed that the region is similar in both strains except for a part encompassing the intergenic region and the N-terminal regions of *SXI1* and *SXI2*, which is highly divergent (Figure 4). This is similar to previous findings in other basidiomycetes, where it was shown that the divergent N-termini of the SXI1 and SXI2 proteins are relevant for the interactions of the two different types of HD transcription factors, and that allele specificity is conferred by these regions (Banham *et al.* 1995; Kämper *et al.* 1995, 2020; Metin *et al.* 2010; Findley *et al.* 2012).

Thus, the two strains carry different and potentially mating-compatible alleles at the *P/R* as well as the *HD* locus, and one can conclude that there are at least two alleles for each *MAT* locus present in the population. Mating between CBS6241 and CBS6242 was observed in 1972 (Rodrigues De Miranda 1972). However, in our laboratories, we were not able to observe sexual structures in co-cultures of the two strains. It is possible that we were not able to recreate the conditions required for mating of the two strains, or that the strains have lost the ability for sexual reproduction through accumulation of mutations after several decades of being cultured under laboratory conditions. The availability of genome sequences for two strains of *F. floriforme* should facilitate the analysis of *MAT* alleles of additional strains of this species that might be used in future mating experiments.

### Characterization of CAZymes repertoire

**Figure 5:**
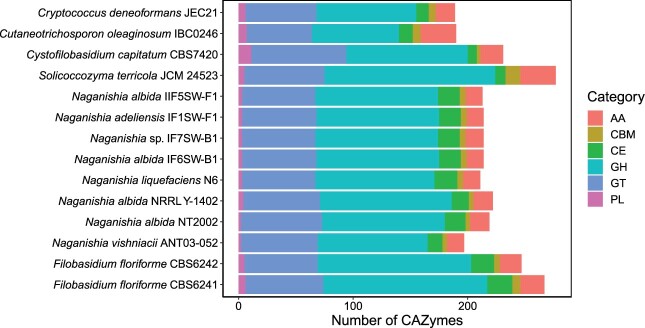
Overview of CAZymes identified in *F. floriforme* compared to other Tremellomycetes. Abbreviations of CAZyme categories: AA, auxiliary activities; CBM, carbohydrate binding modules; CE, carbohydrate esterases; GH, glycoside hydrolases; GT, glycosyltransferases; PL, polysaccharide lyases.

Characterization of CAZymes content revealed that *F. floriforme* contains a high diversity of CAZymes, which includes 267 and 247 genes for CBS6241 and CBS6242, respectively (Figure 5). *Solicoccozyma terricola*, a promising biotechnological strain (Tanimura *et al.* 2014; Close *et al.* 2016) contained the highest number of CAZymes (277), but a similar number (149) of glycoside hydrolases (GHs) compared to the *F. floriforme* strains (143 and 134). *Filobasidium floriforme* contained the highest number of carbohydrate esterases (CEs), while *Cystofilobasidium capitatum* contained the most glycosyltransferases (GTs) and polysaccharide lyases (PLs). *Filobasidium floriforme* comprised 5 CAZymes that were absent in the other organisms (PL3, PL27, GH39, GT28, and GT34) and 8 with a higher number of copies than any of the other species (AA9, CE5, CE9, GH3, GH10, GH31, GH35, and GH43) (Supplementary Figures S5–S10). Interestingly, most of these (AA9, CE5, CE9, GH3, GH10, GH31, GH35, GH39, GH43, GT34, and PL3) are important plant-cell-wall degrading enzymes (Zhao *et al.* 2013; Chang *et al.* 2016). This highlights the promising vast potential of *F. floriforme* in industrial and biotechnological application (Bosetto *et al.* 2016).

## Data availability

PacBio reads from CBS6241 (genome sequencing) were deposited in the NCBI SRA database under accession number SRP256613. Illumina reads from CBS6241 (RNA-seq) were deposited at the NCBI SRA database under accession number SRP256618. Illumina reads from CBS6242 (genome sequencing) were deposited in the NCBI SRA database under accession number SRP258233. The CBS6241 genome sequence (BioProject ID PRJNA621322) was deposited at DDBJ/ENA/GenBank under the accession JAIFAB000000000, and the CBS6242 genome sequence (BioProject ID PRJNA627804) under the accession JABELV000000000. Supplemental material (Supplementary Figures S1–S10, Tables S1–S3, Supplementary Texts S1–S2) is part of the manuscript submission. Supplementary Figure S1: Phylogenetic analysis of Tremellomycetes. Supplementary Figure S2: Multiple alignment of Ste3 homologs in the *MAT* loci of several Tremellomycetes. Supplementary Figure S3: Analysis of Sxi proteins from Tremellomycetes. Supplementary Figure S4: *MAT* loci of CBS6241 and CBS6242. Supplementary Figure S5: Overview of CAZyme category AA. Supplementary Figure S6: Overview of CAZyme category CBM. Supplementary Figure S7: Overview of CAZyme category CE. Supplementary Figure S8: Overview of CAZyme category GH. Supplementary Figure S9: Overview of CAZyme category GT. Supplementary Figure S10: Overview of CAZyme category PL. Supplementary Table S1: Genome assemblies that were used in this study. Supplementary Table S2: Analysis of putative telomeric repeats at contig ends of CBS6241. Supplementary Table S3: Analysis of unique orthogroups in *F. floriforme*. Supplementary Text S1: Perl script for searching for putative pheromone genes. Supplementary Text S2: Perl script for searching for putative telomeric repeats.

Supplementary material is available at *G3* online.

## Supplementary Material

jkab398_Supplementary_Data
