# Supplementary material for: Draft genome sequences of strains CBS6241 and CBS6242 of the basidiomycetous yeast Filobasidium floriforme
Source: G3 (Bethesda). 2021 Nov 15;12(2):jkab398. doi: 10.1093/g3journal/jkab398 (PMC9210288; doi:10.1093/g3journal/jkab398)
Supplement: jkab398_Supplementary_Data [file jkab398_supplementary_data.pdf]

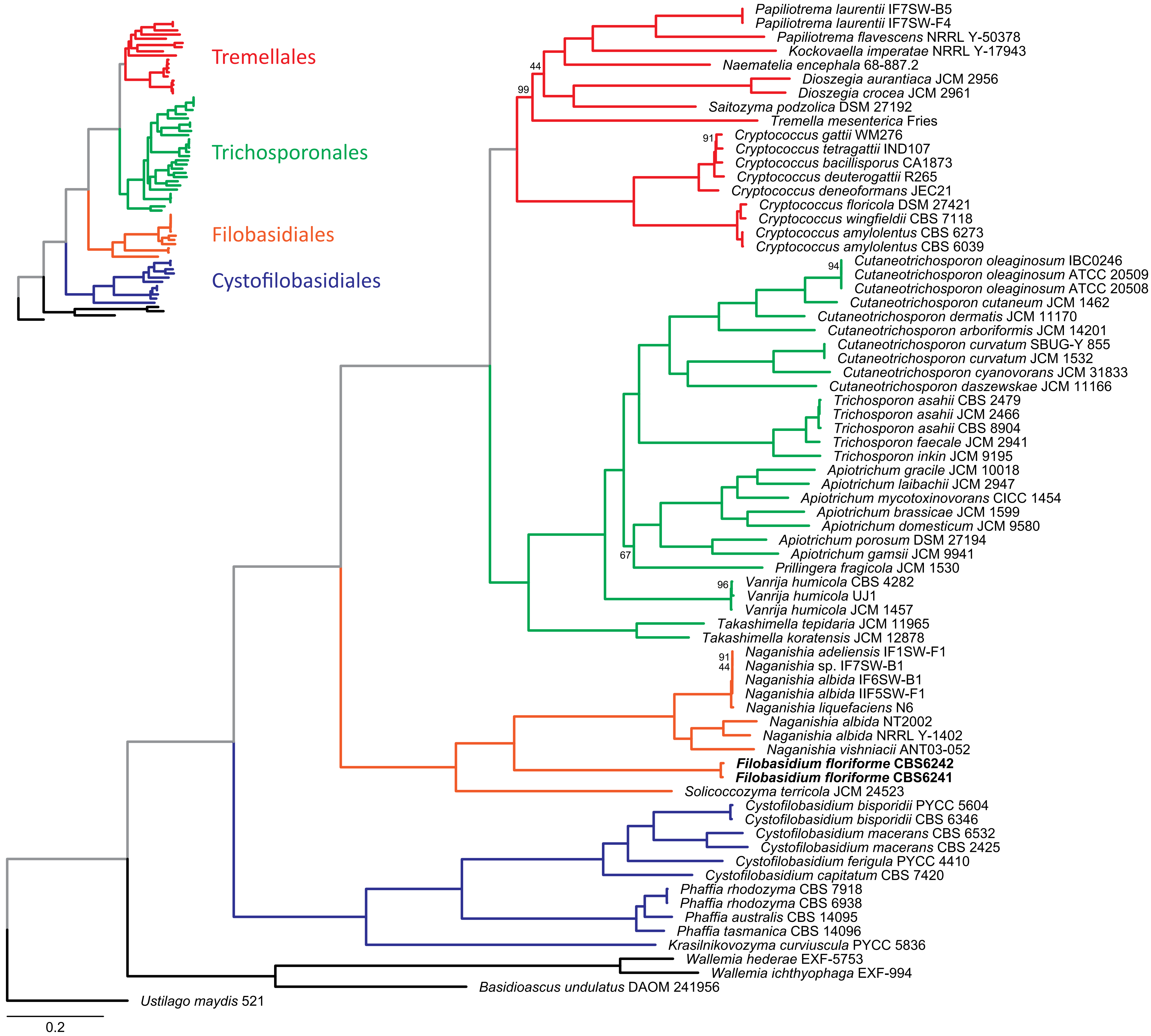

Figure S1. Phylogenetic analysis of Tremellomycetes. A maximum likelihood analysis of 142 single-copy orthologous protein sequences was performed with 500 bootstrap replicates using *Ustilago maydis* as an outgroup. Bootstrap values are 100 % except where lower values are indicated at the branches. The scale bar gives substitutions per site. Accession numbers for the genome assembly data are provided in Table S1.

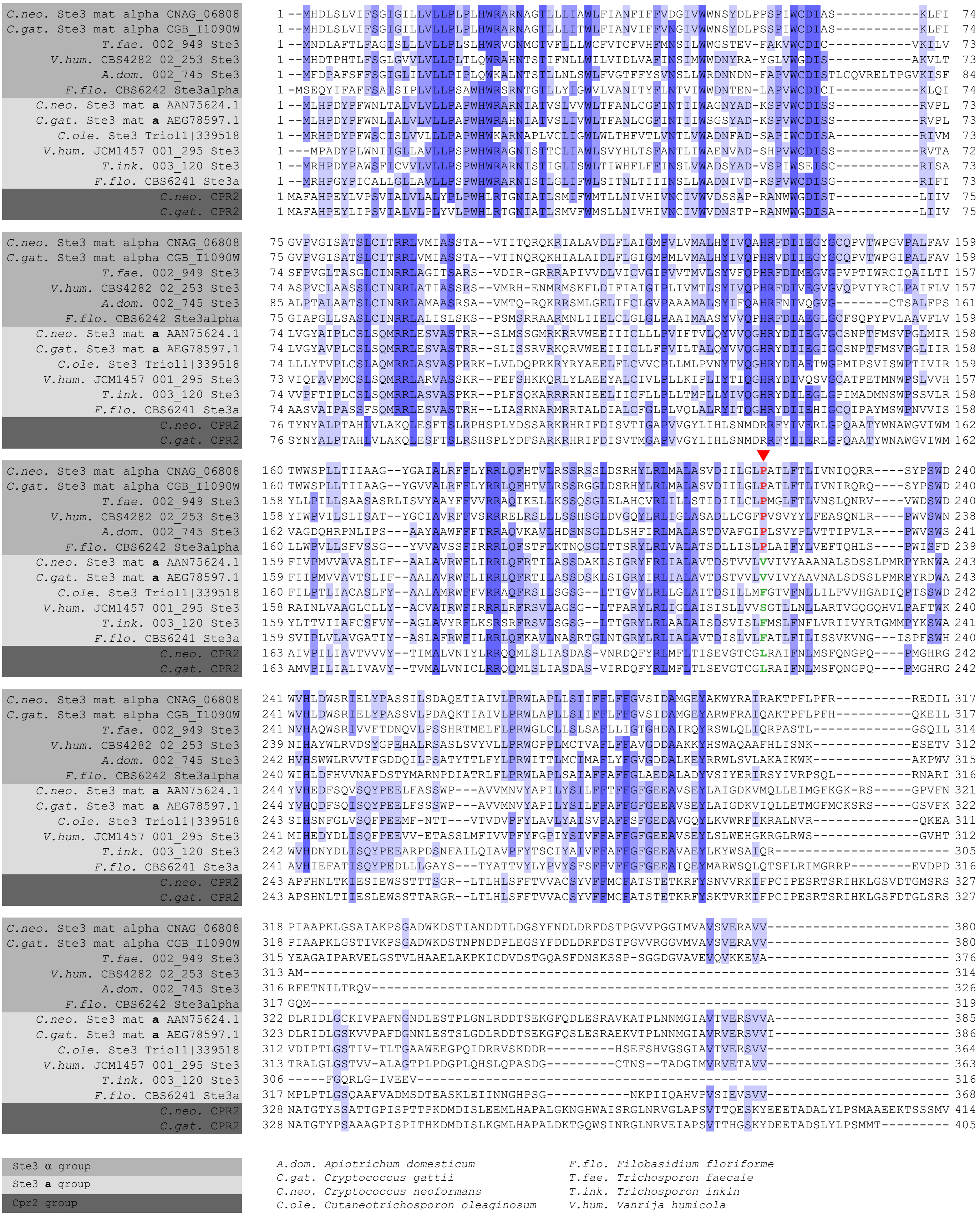

Figure S2. Multiple alignment of Ste3 homologs in the *MAT* loci of several *Tremellomycetes*. A conserved proline residue that is present in the α group proteins is indicated in red. It is not present in the **a**-specific Ste3 proteins from *Tremellomycetes*. In the constitutively active pheromone receptor-like Cpr2 protein, this residue is changed to leucine (Y.P. Hsueh, C. Xue, and J. Heitman, EMBO J 28:1220-1233). The *F. floriforme* Ste3 proteins belong to the Ste3**a** (CBS6241) and Ste3α (CBS6242) groups. Accession numbers or locus tag numbers: *A.dom.*, T.domesticum_002_745 (genome accession BCFW01000000), *C.neo.*, Ste3a: AAN75624.1, Ste3α: XP_012049557.1, Cpr2: XP_012047561.1; *C.gat.*, Ste3a: AEG78597.1, Ste3α: XP_003196044.1, Cpr2: XP_003191200.1; *C.ole.*, XP_018276494.1, *F.flo.*, CBS6241: gene_1555, CBS6242: CBS6242_07693= FFLO_06159; *T.fae.*, T.faecale_002_949 (genome accession JXYK01000000); *T.ink.*, T.inkin_003_120 (genome accession JXYM01000000); *V.hum.*, Ste3a: JCM1475_001_295 (genome accession BCJF01000000), Ste3α: TXT13458.1.

A

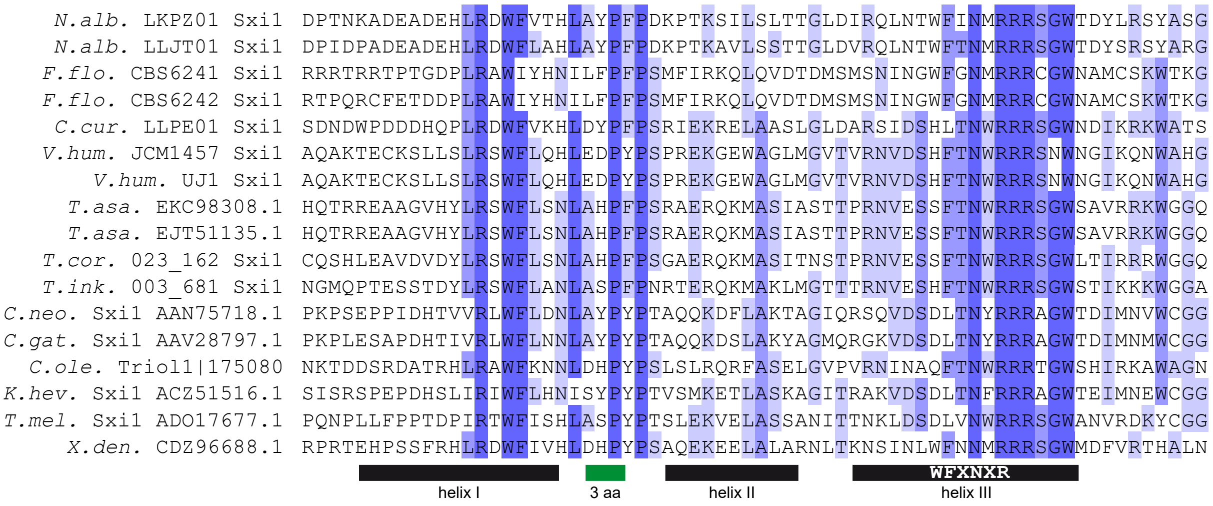

B

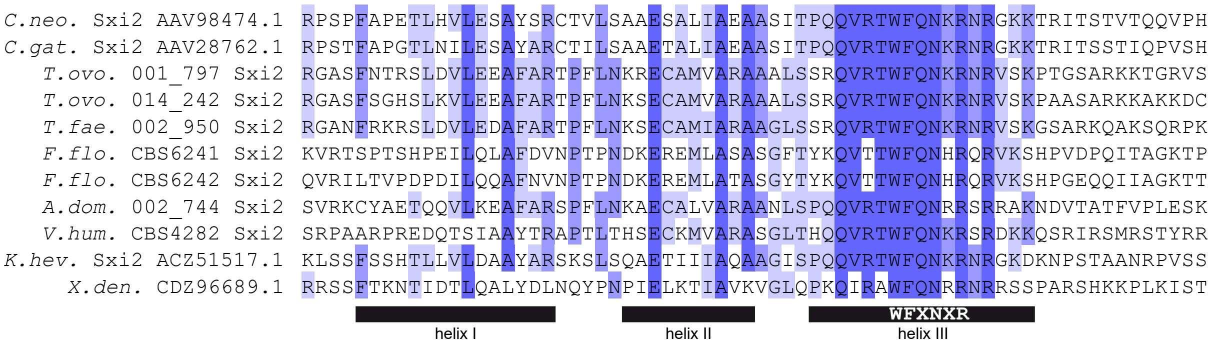

Figure S3. Analysis of Sxi proteins from *Tremellomycetes*. **A.** Multiple alignment of the homeodomains from Sxi1 homologs (class HD1 homeodomain transcription factors) from *Tremellomycetes*. The three conserved helices are underlined in black. A three amino acid insertion characteristic for the HD1 homeodomain factors (in contrast to the HD2 homeodomains) is underlined in green. The conserved DNA binding motif WFXNXR within helix III is indicated. Labelling of helices and motifs according to Kües et al. (The Mycota XIV, 2011, p. 97-160. Springer, Berlin, Heidelberg). Species: *C.neo. Cryptococcus neoformans, C.gat. Cryptococcus gattii, C.cur. Cutaneotrichosporon curvatus, C.ole. Cutaneotrichosporon oleaginosum, F.flo. Filobasidium floriforme*, *K.hev. Kwoniella heveanensis, N.alb. Naganishia albida, T.asa. Trichosporon asahii, T.cor. Trichosporon coremiiforme, T.ink. Trichosporon inkin, T.mel. Tremella mesenterica, V.hum. Vanrija humicola, X.den. Xanthophyllomyces dendrorhous*. **B.** Multiple alignment of the homeodomains from Sxi2 homologs (class HD2 homeodomain transcription factors) from *Tremellomycetes*. Labelling and species names as in A with addition of *A.dom. Apiotrichum domesticum, T.fae. Trichosporon faecale, T.ovo. Trichosporon ovoides*.

GenBank accession numbers are given for the *C. neoformans*, *C. gattii*, *K. heveanensis*, *T. asahii*, *T. mesenterica*, and *X. dendrorhous* proteins. For the other species, HD proteins were from predicted genes or manually annotated in the following genome sequences: *A. domesticum* BCFW01000000, *C. curvatus* LDEP01000000, *C. oleaginosum* JZUH00000000, *F. floriforme* (this study), *N. albida* LKPZ00000000.1 and LLJT01000000, *T. coremiiforme* JXYL01000000, *T. inkin* JXYM01000000, *V. humicola* BFAH01000000 and QKWK01000000, *T. faecale* JXYK01000000, *T. ovoides* JXYN01000000.

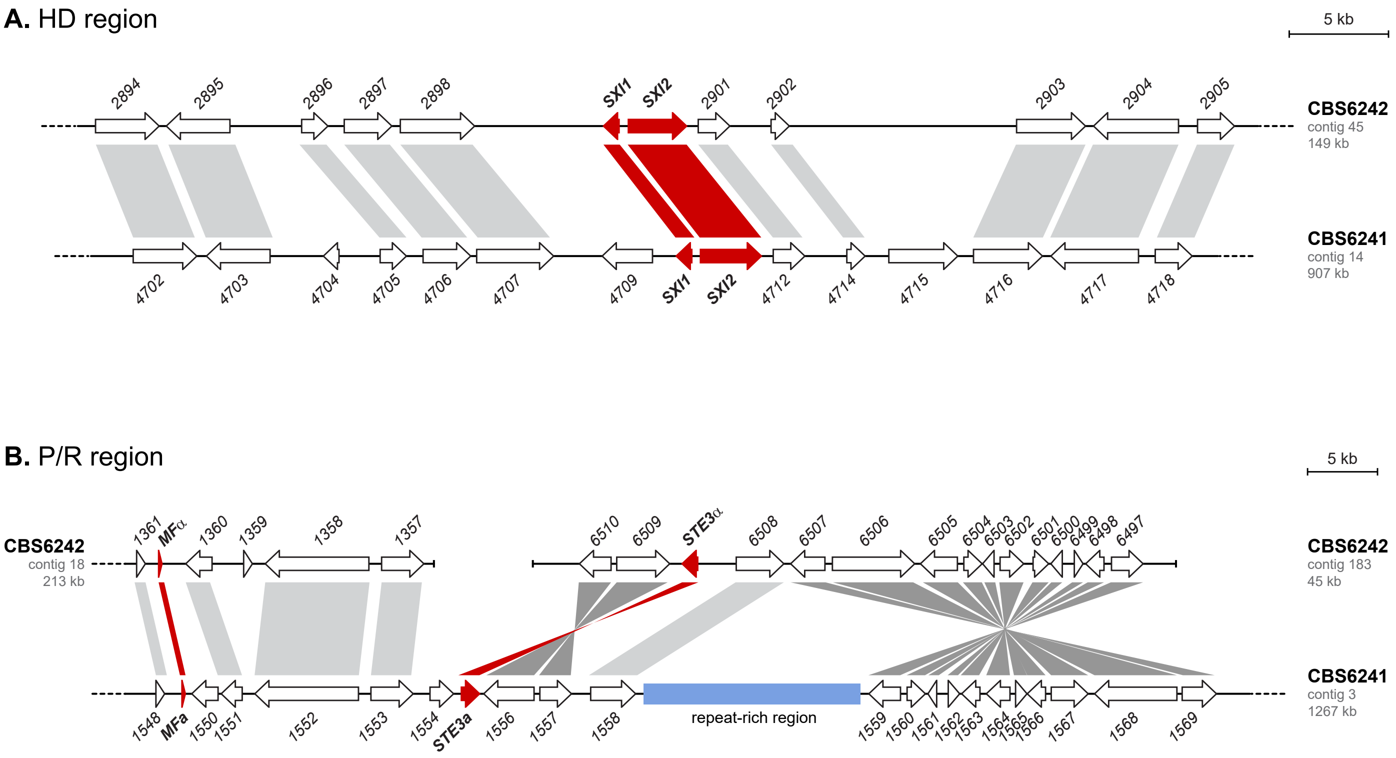

Figure S4. *MAT* loci of CBS6241 and CBS6242. The mating-type defining genes (*SXI1*, *SXI2*, *STE3*, and the pheromone precursor genes *MF**a*** and *MF*α) are shown in red, other genes in white. A 15 kb repeat-rich region present in the P/R region of CBS6241 is shown in blue. Orthologs (identified by bi-directional BLAST analysis) are connected by light grey or dark grey bars when in the same or opposite orientation, respectively.

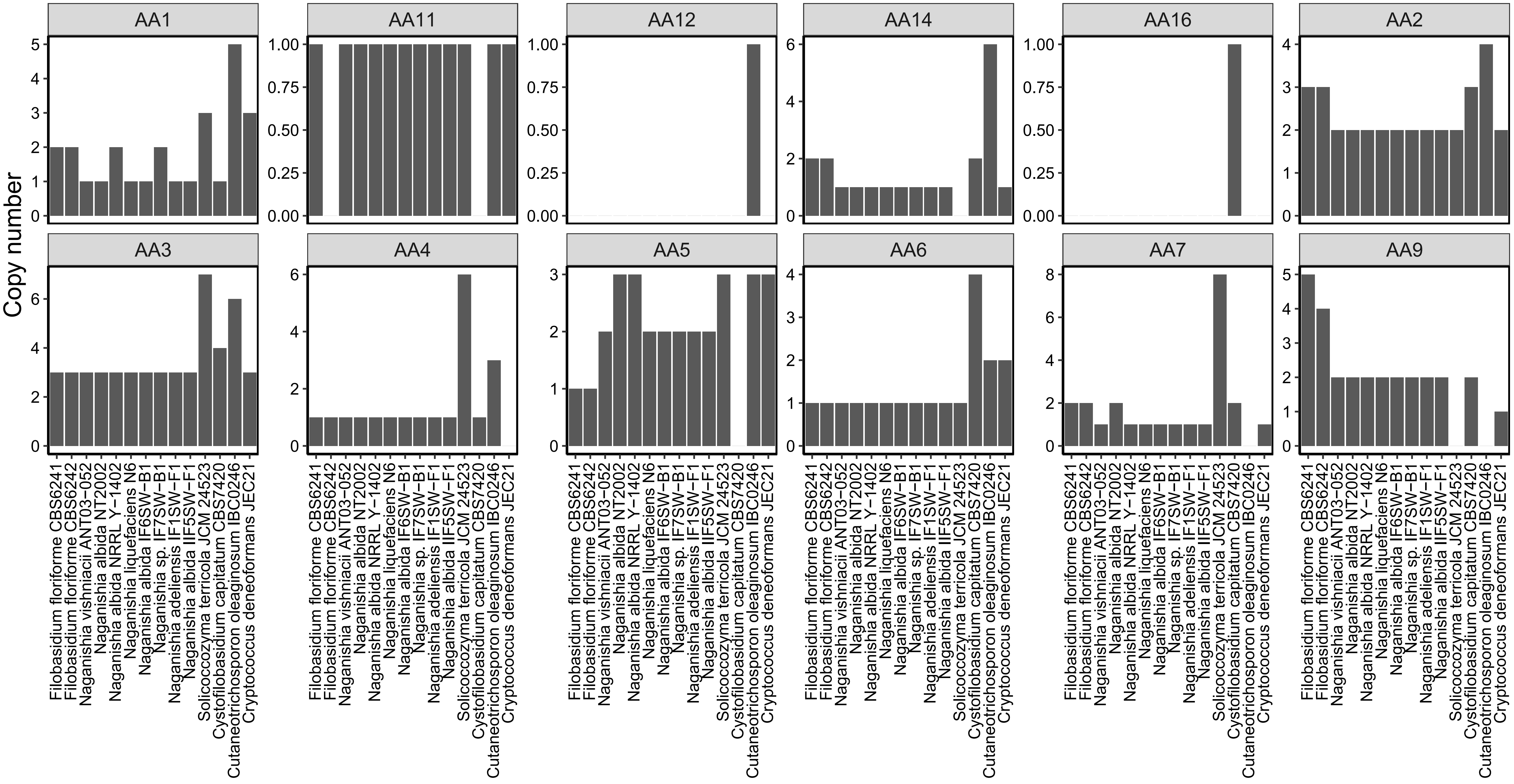

Figure S5. Overview of CAZyme category AA (auxiliary activities).

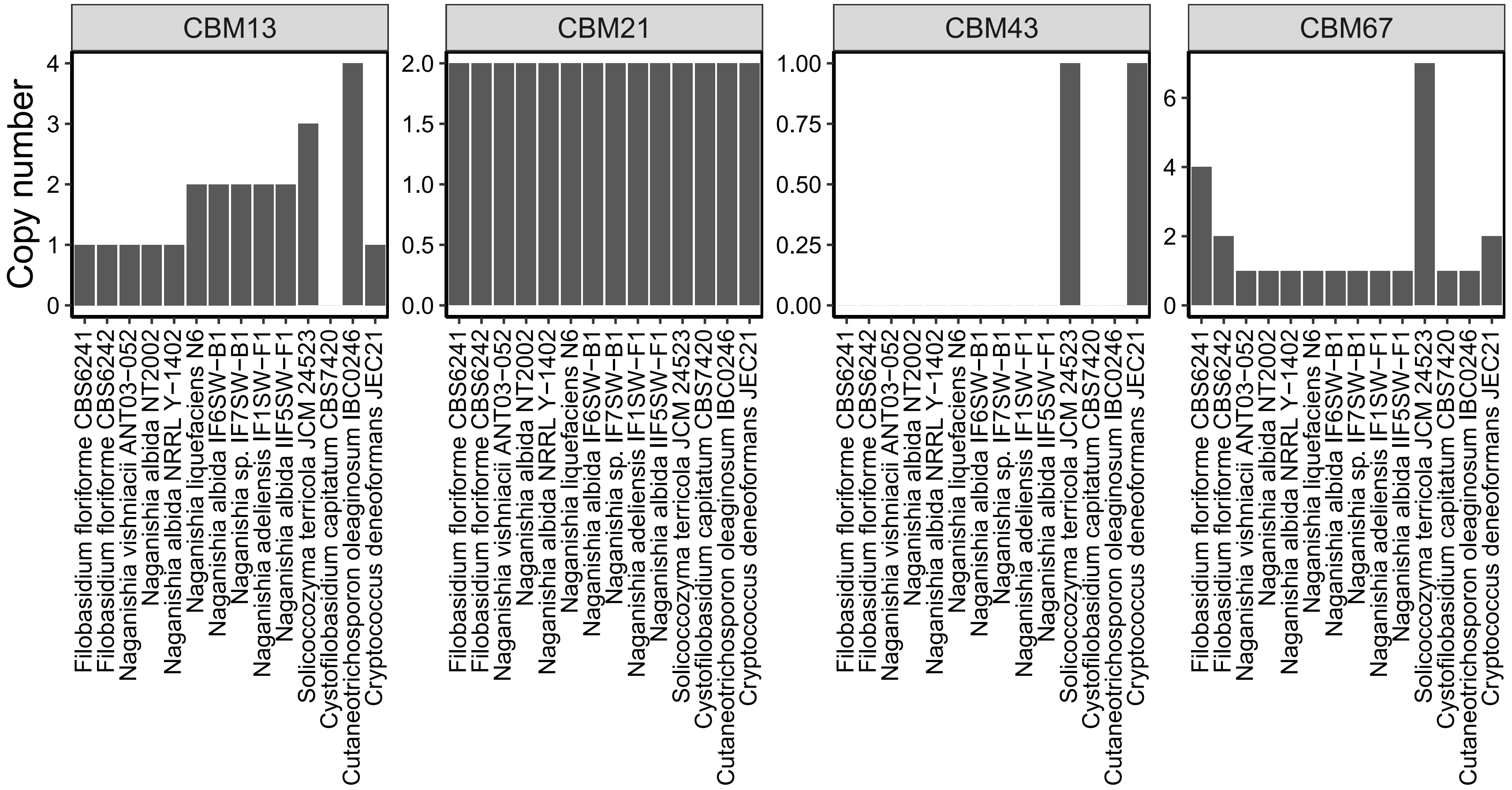

Figure S6. Overview of CAZyme category CBM (carbohydrate binding modules).

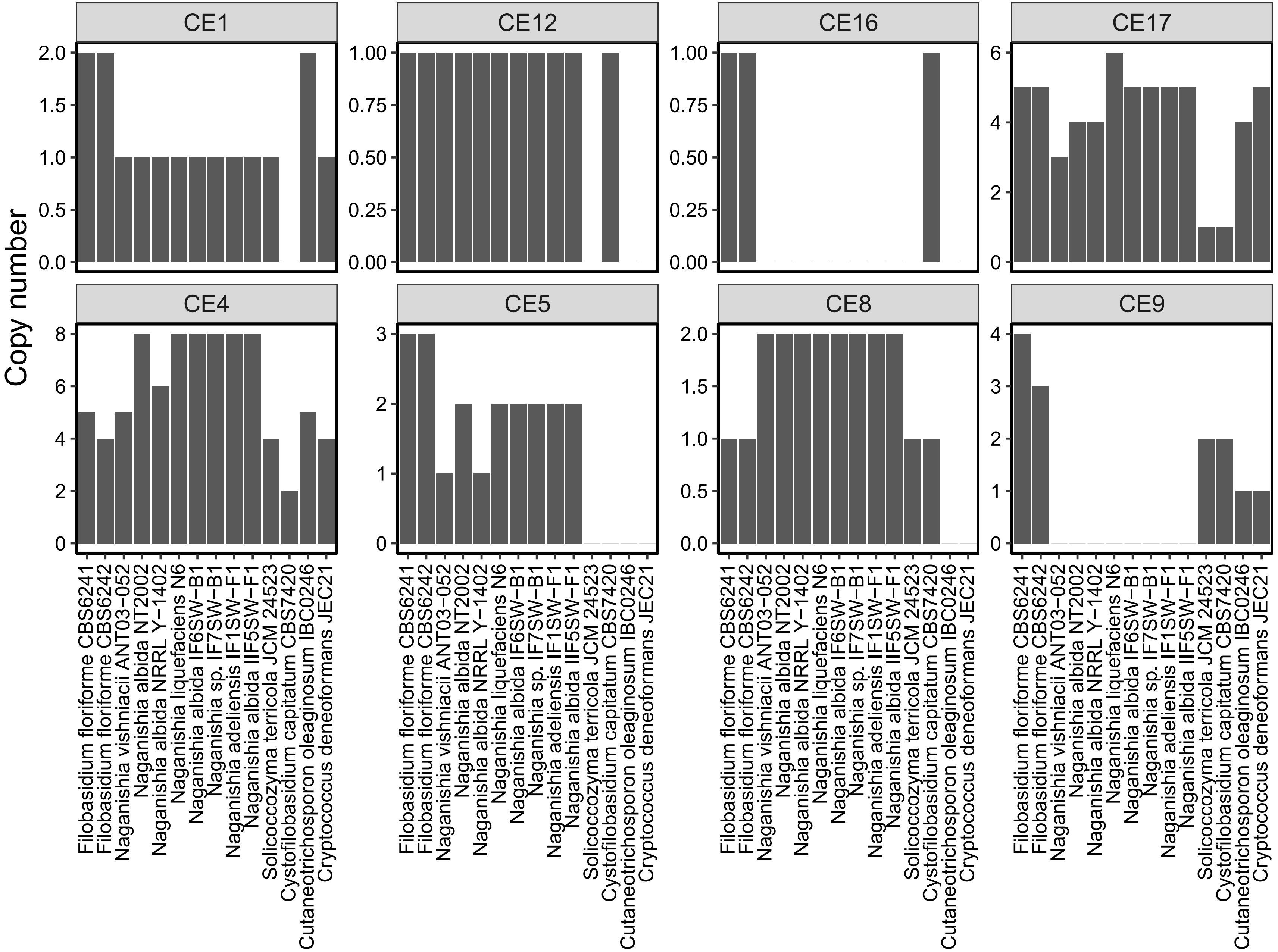

Figure S7. Overview of CAZyme category CE (carbohydrate esterases).

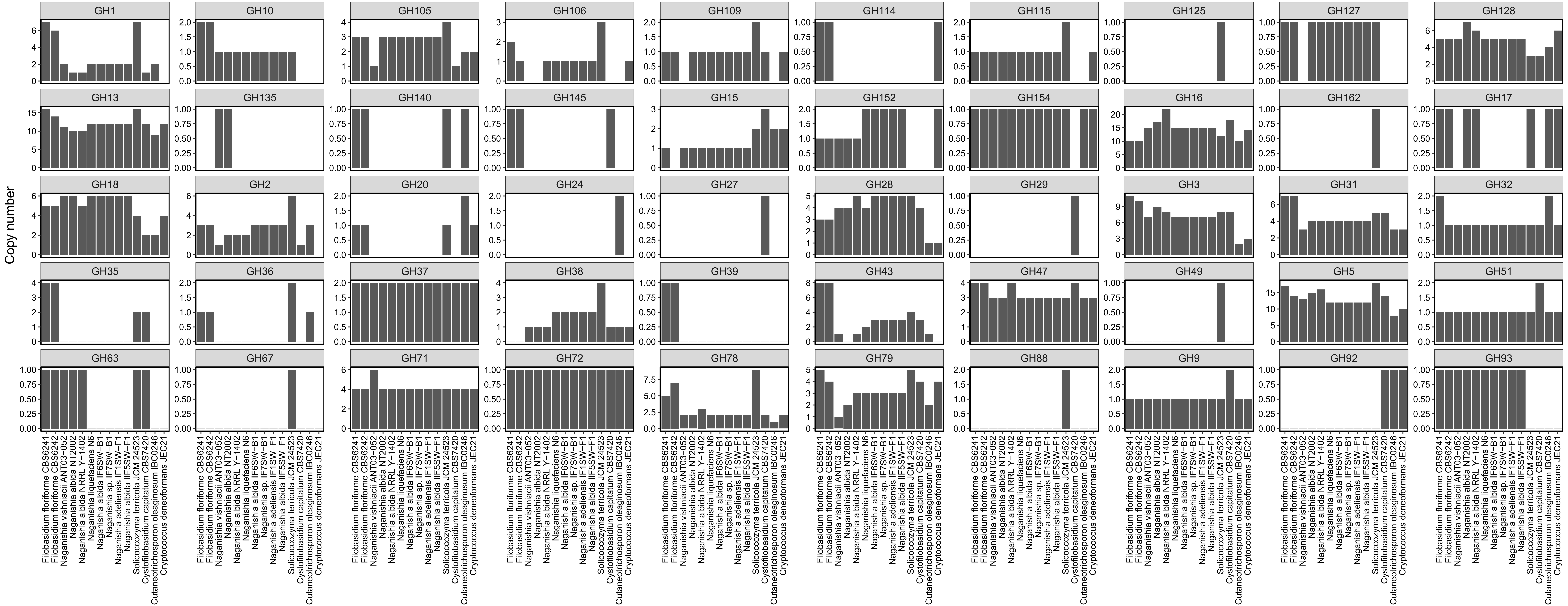

Figure S8. Overview of CAZyme category GH (glycoside hydrolases).

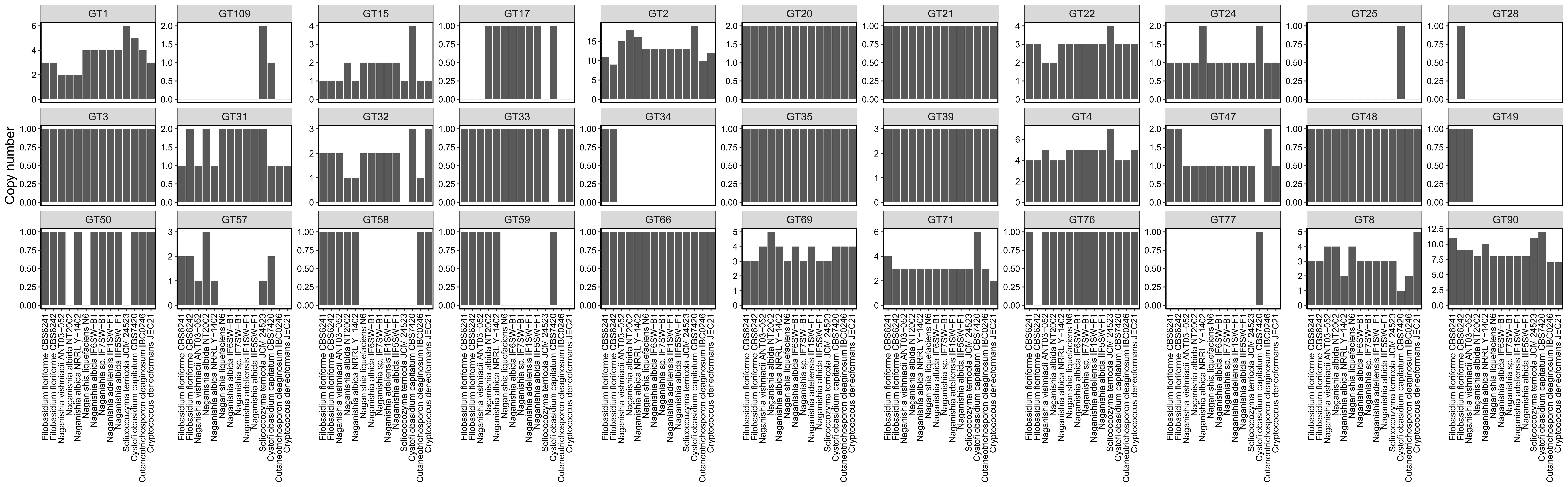

Figure S9. Overview of CAZyme category GT (glycosyltransferases).

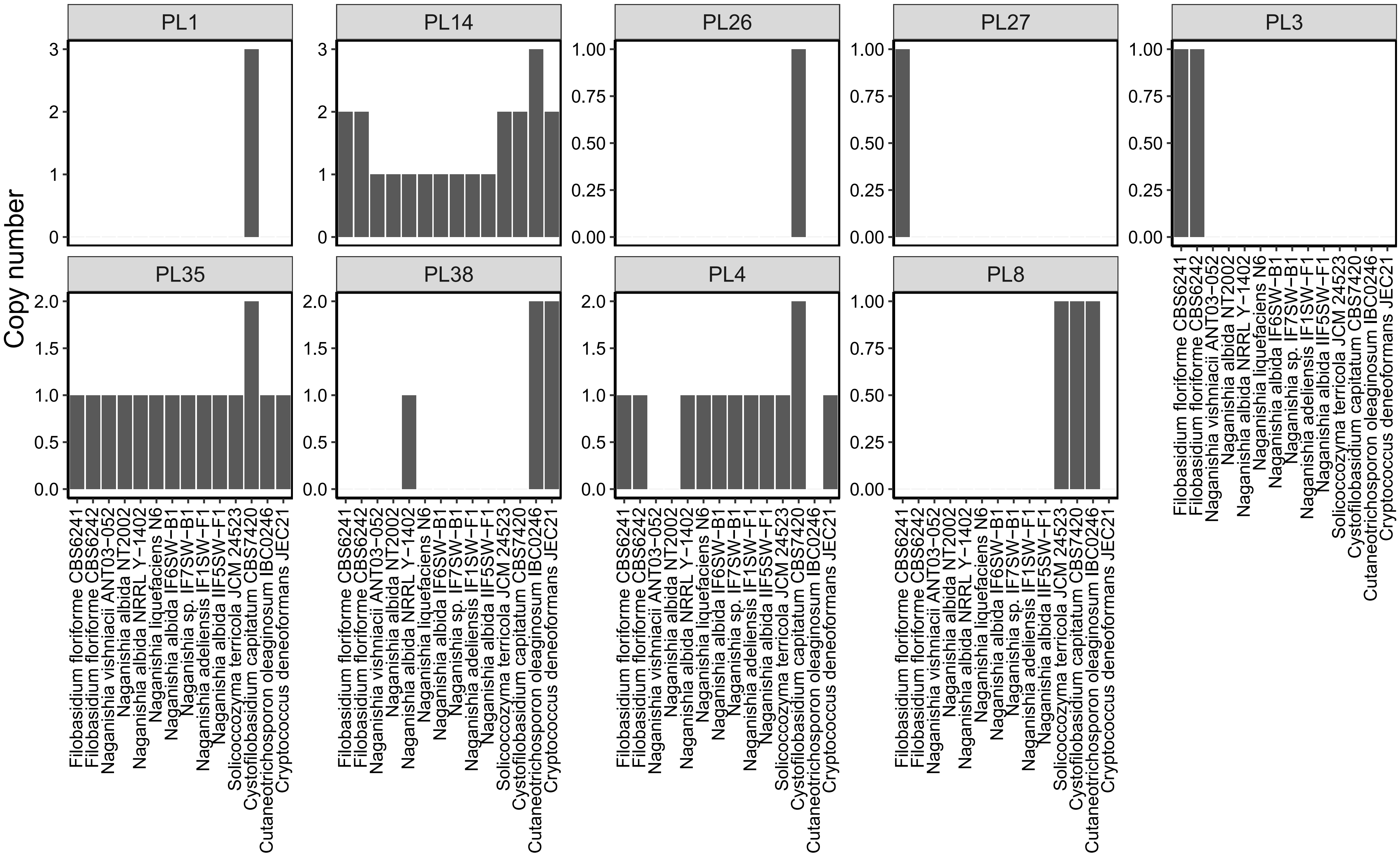

Figure S10. Overview of CAZyme category PL (polysaccharide lyases).

Table S1: Genome assemblies that were used in this study

Order	Genus	Species	Strain	Assembly	Reference
Filobasidiales	Filobasidium	*Filobasidium floriforme*	CBS6241	JGI v1.0	This study
Filobasidiales	Filobasidium	*Filobasidium floriforme*	CBS6242	GCA_019201745.1	This study
Filobasidiales	Naganishia	*Naganishia adeliensis*	IF1SW-F1	GCA_012922715.1	Bijlani et al. 2020
Filobasidiales	Naganishia	*Naganishia albida*	IF6SW-B1	GCA_012922605.1	Bijlani et al. 2020
Filobasidiales	Naganishia	*Naganishia albida*	IIF5SW-F1	GCA_012922635.1	Bijlani et al. 2020
Filobasidiales	Naganishia	*Naganishia albida*	NRRL Y-1402	GCA_001444555.1	Vajpeyi and Chandran 2016
Filobasidiales	Naganishia	*Naganishia albida*	NT2002	GCA_001468955.1	Yong et al. 2016
Filobasidiales	Naganishia	*Naganishia liquefaciens*	N6	GCA_013423385.1	Han et al. 2020
Filobasidiales	Naganishia	*Naganishia sp.*	IF7SW-B1	GCA_012922685.1	Bijlani et al. 2020
Filobasidiales	Naganishia	*Naganishia vishniacii*	ANT03-052	GCA_015708705.1	Nizovoy et al. 2021
Filobasidiales	Solicoccozyma	*Solicoccozyma terricola*	JCM 24523	GCA_001712455.1	Close et al. 2016a
Tremellales	Cryptococcus	*Cryptococcus amylolentus*	CBS 6039	GCF_001720205.1	Sun et al. 2017
Tremellales	Cryptococcus	*Cryptococcus amylolentus*	CBS 6273	GCA_001720235.1	Sun et al. 2017
Tremellales	Cryptococcus	*Cryptococcus bacillisporus*	CA1873	GCA_000855695.1	Farrer et al. 2015
Tremellales	Cryptococcus	*Cryptococcus deneoformans*	JEC21	GCF_000091045.1	Loftus *et al.* 2005
Tremellales	Cryptococcus	*Cryptococcus deuterogattii*	R265	GCA_003011995.1	D'Souza et al. 2011
Tremellales	Cryptococcus	*Cryptococcus floricola*	DSM 27421	GCA_006352305.1	Passer et al. 2019
Tremellales	Cryptococcus	*Cryptococcus gattii*	WM276	GCF_000185945.1	D'Souza et al. 2011
Tremellales	Cryptococcus	*Cryptococcus tetragattii*	IND107	GCA_000835755.1	Farrer et al. 2015
Tremellales	Cryptococcus	*Cryptococcus wingfieldii*	CBS 7118	GCA_006149155.1	Passer et al. 2019
Tremellales	Dioszegia	*Dioszegia aurantiaca*	JCM 2956	GCA_001600655.1	Takashima et al. 2019
Tremellales	Dioszegia	*Dioszegia crocea*	JCM 2961	GCA_001600615.1	Takashima et al. 2019
Tremellales	Kockovaella	*Kockovaella imperatae*	NRRL Y-17943	GCF_002102565.1	Mondo et al. 2017
Tremellales	Naematelia	*Naematelia encephala*	68-887.2	GCA_002105065.1	Mondo et al. 2017
Tremellales	Papiliotrema	*Papiliotrema flavescens*	NRRL Y-50378	GCA_000442785.1	Rong and Gardener 2013
Tremellales	Papiliotrema	*Papiliotrema laurentii*	IF7SW-B5	GCA_012922625.1	Bijlani et al. 2020
Tremellales	Papiliotrema	*Papiliotrema laurentii*	IF7SW-F4	GCA_012922615.1	Bijlani et al. 2020
Tremellales	Saitozyma	*Saitozyma podzolica*	DSM 27192	GCA_003942215.1	Aliyu et al. 2019
Tremellales	Tremella	*Tremella mesenterica*	Fries	GCF_000271645.1	Floudas et al. 2012
Trichosporonales	Apiotrichum	*Apiotrichum brassicae*	JCM 1599	GCA_001600295.1	Takashima et al. 2015

Trichosporonales	Apiotrichum	*Apiotrichum domesticum*	JCM 9580	GCA_001599015.1	Cho et al. 2016
Trichosporonales	Apiotrichum	*Apiotrichum gamsii*	JCM 9941	GCA_001600315.1	Takashima et al. 2015
Trichosporonales	Apiotrichum	*Apiotrichum gracile*	JCM 10018	GCA_001600335.1	Takashima et al. 2015
Trichosporonales	Apiotrichum	*Apiotrichum laibachii*	JCM 2947	GCA_001600735.1	Takashima et al. 2015
Trichosporonales	Apiotrichum	*Apiotrichum mycotoxinovorans*	CICC 1454	GCA_013177335.1	Sun et al. 2020
Trichosporonales	Apiotrichum	*Apiotrichum porosum*	DSM 27194	GCF_003942205.1	Gorte et al. 2019
Trichosporonales	Cutaneotrichosporon	*Cutaneotrichosporon arboriformis*	JCM 14201	GCA_002335565.1	Takashima et al. 2018
Trichosporonales	Cutaneotrichosporon	*Cutaneotrichosporon curvatum*	JCM 1532	GCA_001600275.1	Takashima et al. 2015
Trichosporonales	Cutaneotrichosporon	*Cutaneotrichosporon curvatum*	SBUG-Y 855	GCA_001028165.1	Hofmeyer et al. 2016
Trichosporonales	Cutaneotrichosporon	*Cutaneotrichosporon cutaneum*	JCM 1462	GCA_001600715.1	Takashima et al. 2015
Trichosporonales	Cutaneotrichosporon	*Cutaneotrichosporon cyanovorans*	JCM 31833	GCA_002335625.1	Takashima et al. 2018
Trichosporonales	Cutaneotrichosporon	*Cutaneotrichosporon daszewskae*	JCM 11166	GCA_002335585.1	Takashima et al. 2018
Trichosporonales	Cutaneotrichosporon	*Cutaneotrichosporon dermatis*	JCM 11170	GCA_003116895.1	Takashima et al. 2018
Trichosporonales	Cutaneotrichosporon	*Cutaneotrichosporon oleaginosum*	ATCC 20508	GCA_008065305.1	Sun et al. 2019
Trichosporonales	Cutaneotrichosporon	*Cutaneotrichosporon oleaginosum*	ATCC 20509	JGI v1.0	Close et al. 2016b
Trichosporonales	Cutaneotrichosporon	*Cutaneotrichosporon oleaginosum*	IBC0246	GCF_001027345.1	Kourist *et al.* 2015
Trichosporonales	Prillingera	*Prillingera fragicola*	JCM 1530	GCA_002335605.1	Takashima et al. 2018
Trichosporonales	Takashimella	*Takashimella koratensis*	JCM 12878	GCA_003116875.1	Takashima et al. 2018
Trichosporonales	Takashimella	*Takashimella tepidaria*	JCM 11965	GCA_003116915.1	Takashima et al. 2018
Trichosporonales	Trichosporon	*Trichosporon asahii*	CBS 2479	GCF_000293215.1	Yang et al. 2012a
Trichosporonales	Trichosporon	*Trichosporon asahii*	CBS 8904	GCA_000299215.2	Yang et al. 2012b
Trichosporonales	Trichosporon	*Trichosporon asahii*	JCM 2466	GCA_001972365.1	Takashima et al. 2015
Trichosporonales	Trichosporon	*Trichosporon coremiiforme*	JCM 2938	JXYL00000000.1	Sriswasdi et al. 2016
Trichosporonales	Trichosporon	*Trichosporon faecale*	JCM 2941	GCA_001752585.1	Sriswasdi et al. 2016
Trichosporonales	Trichosporon	*Trichosporon inkin*	JCM 9195	GCA_001752625.1	Sriswasdi et al. 2016
Trichosporonales	Trichosporon	*Trichosporon ovoides*	JCM 9940	JXYN00000000.1	Sriswasdi et al. 2016
Trichosporonales	Vanrija	*Vanrija humicola*	CBS 4282	GCA_008065275.1	Sun et al. 2019
Trichosporonales	Vanrija	*Vanrija humicola*	JCM 1457	GCA_001600235.1	Takashima et al. 2015
Trichosporonales	Vanrija	*Vanrija humicola*	UJ1	GCA_002897395.1	Imanishi et al. 2018
Cystofilobasidiales	Cystofilobasidium	*Cystofilobasidium bisporidii*	CBS 6346	GCA_014825555.1	David-Palma et al. 2020
Cystofilobasidiales	Cystofilobasidium	*Cystofilobasidium bisporidii*	PYCC 5604	GCA_014825535.1	David-Palma et al. 2020
Cystofilobasidiales	Cystofilobasidium	*Cystofilobasidium capitatum*	CBS7420	GCA_014825545.1	David-Palma et al. 2020
Cystofilobasidiales	Cystofilobasidium	*Cystofilobasidium ferigula*	PYCC 4410	GCA_014825675.1	David-Palma et al. 2020

Cystofilobasidiales	Cystofilobasidium	*Cystofilobasidium macerans*	CBS 2425	GCA_014825765.1	David-Palma et al. 2020
Cystofilobasidiales	Cystofilobasidium	*Cystofilobasidium macerans*	CBS 6532	GCA_014825745.1	David-Palma et al. 2020
Cystofilobasidiales	Krasilnikovozyma	*Krasilnikovozyma curviuscula*	PYCC 5836	GCA_014825775.1	David-Palma et al. 2020
Cystofilobasidiales	Mrakia	*Mrakia blollopis*	SK-4	GCA_000950635.1	Tsuji et al. 2015
Cystofilobasidiales	Mrakia	*Mrakia psychrophila*	NN053900	GCA_001889225.1	Su et al. 2016
Cystofilobasidiales	Phaffia	*Phaffia australis*	CBS 14095	GCA_014825575.1	David-Palma et al. 2020
Cystofilobasidiales	Phaffia	*Phaffia rhodozyma*	CBS 6938	GCA_014706385.1	Sharma et al. 2015
Cystofilobasidiales	Phaffia	*Phaffia rhodozyma*	CBS 7918	GCA_001579715.1	Bellora et al. 2016
Cystofilobasidiales	Phaffia	*Phaffia tasmanica*	CBS 14096	GCA_014825565.1	David-Palma et al. 2020
Ustilaginales	Ustilago	*Ustilago maydis*	521	GCF_000328475.2	Kämper et al. 2006
Wallemiales	Basidioascus	*Basidioascus undulatus*	DAOM 241956	GCA_000826855.1	Nguyen et al. 2015
Wallemiales	Wallemia	*Wallemia hederae*	EXF-5753	GCA_004918325.1	Gostinčar et al. 2019
Wallemiales	Wallemia	*Wallemia ichthyophaga*	EXF-994	GCF_000400465.1	Zajc et al. 2013

References for Table S1

Aliyu H, Gorte O, Zhou X, Neumann A, Ochsenreither K (2020) In silico proteomic analysis provides insights into phylogenomics and plant biomass deconstruction potentials of the Tremelalles. Front Bioeng Biotechnol 8:226

Bellora N, Moliné M, David-Palma M, Coelho MA, Hittinger CT, Sampaio JP, Gonçalves P, Libkind D (2016) Comparative genomics provides new insights into the diversity, physiology, and sexuality of the only industrially exploited tremellomycete: *Phaffia rhodozyma* . BMC Genomics 17:901

Bijlani S, Singh NK, Mason CE, Wang CCC, Venkateswaran K (2020) Draft genome sequences of Tremellomycetes strains isolated from the International Space Station. Microbiol Resour Announc 9:e00504-20

Cho O, Ichikawa T, Kurakado S, Takashima M, Manabe R, Ohkuma M, Sugita T (2016) Draft genome sequence of the causative antigen of summer-type hypersensitivity pneumonitis, *Trichosporon domesticum* JCM 9580. Genome Announc 4:e00651-16

Close D, Ojumu J, Zhang G (2016) Draft genome sequence of *Cryptococcus terricola* JCM 24523, an oleaginous yeast capable of expressing exogenous DNA. Genome Announc 4:e01238-16

Close D, Ojumu J (2016) Draft genome sequence of the oleaginous yeast *Cryptococcus curvatus* ATCC 20509. Genome Announc 4:e01235-16

D'Souza CA, Kronstad JW, Taylor G, Warren R, Yuen M, Hu G, Jung WH, Sham A, Kidd SE, Tangen K, Lee N, Zeilmaker T, Sawkins J, McVicker G, Shah S, Gnerre S, Griggs A, Zeng Q, Bartlett K, Li W, Wang X, Heitman J, Stajich JE, Fraser JA, Meyer W, Carter D, Schein J, Krzywinski M, Kwon-Chung KJ, Varma A, Wang J, Brunham R, Fyfe M, Ouellette BFF, Siddiqui A, Marra M, Jones S, Holt R, Birren BW, Galagan JE, Cuomo CA (2011) Genome variation in *Cryptococcus gattii* , an emerging pathogen of immunocompetent hosts. mBio 2:e00342-10

David-Palma M, Libkind D, Brito PH, Silva M, Bellora N, Coelho MA, Heitman J, Gonçalves P, Sampaio JP (2020) The untapped Australasian diversity of astaxanthin-producing yeasts with biotechnological potential- *Phaffia australis* sp. nov. and *Phaffia tasmanica* sp. nov. Microorganisms 8:1651

Farrer RA, Desjardins CA, Sakthikumar S, Gujja S, Saif S, Zeng Q, Chen Y, Voelz K, Heitman J, May RC, Fisher MC, Cuomo CA (2015) Genome evolution and innovation across the four major lineages of *Cryptococcus gattii* . mBio 6:e00868-15

Floudas D, Binder M, Riley R, Barry K, Blanchette RA, Henrissat B, Martínez AT, Otillar R, Spatafora JW, Yadav JS, Aerts A, Benoit I, Boyd A, Carlson A, Copeland A, Coutinho PM, de Vries RP, Ferreira P, Findley K, Foster B, Gaskell J, Glotzer D, Górecki P, Heitman J, Hesse C, Hori C, Igarashi K, Jurgens JA, Kallen N, Kersten P, Kohler A, Kües U, Kumar TKA, Kuo A, LaButti K, Larrondo LF, Lindquist E, Ling A, Lombard V, Lucas S, Lundell T, Martin R, McLaughlin DJ, Morgenstern I, Morin E, Murat C, Nagy LG, Nolan M, Ohm RA, Patyshakuliyeva A, Rokas A, Ruiz-Dueñas FJ, Sabat G, Salamov A, Samejima M, Schmutz J, Slot JC, St. John F, Stenlid J, Sun H, Sun S, Syed K, Tsang A, Wiebenga A, Young D, Pisabarro A, Eastwood DC, Martin F, Cullen D, Grigoriev IV, Hibbett DS (2012) The paleozoic origin of enzymatic lignin decomposition reconstructed from 31 fungal genomes. Science 336:1715-1719

Gorte O, Aliyu H, Neumann A, Ochsenreither K (2019) Draft genome sequence of the oleaginous yeast *Apiotrichum porosum* (syn. *Trichosporon porosum*) DSM27194. J Genomics 7:11-13

Gostinčar C, Sun X, Zajc J, Fang C, Hou Y, Luo Y, Gunde-Cimerman N, Song Z (2019) Population genomics of an obligately halophilic basidiomycete Wallemia ichthyophaga. Front Microbiol 10:2019

Han YW, Kajitani R, Morimoto H, Palihati M, Kurokawa Y, Ryusui R, Argunhan B, Tsubouchi H, Abe F, Kajiwara S, Iwasaki H, Itoh T (2020) Draft genome sequence of *Naganishia liquefaciens* strain N6, isolated from the Japan Trench. Microbiol Resour Announc 9:e00827-20

Hofmeyer T, Hackenschmidt S, Nadler F, Thürmer A, Daniel R, Kabisch J (2016) Draft genome sequence of *Cutaneotrichosporon curvatus* DSM 101032 (formerly *Cryptococcus curvatus*), an oleaginous yeast producing polyunsaturated fatty acids. Genome Announc 4:e00362-16

Imanishi D, Abe K, Kera Y, Takahashia S (2018) Draft genome sequence of the yeast *Vanrija humicola* (formerly *Cryptococcus humicola*) strain UJ1, a producer of D-aspartate oxidase. Genome Announc 6:e00068-18

Kämper J, Kahmann R, Bölker M, Ma LJ, Brefort T, Saville BJ, Banuett F, Kronstad JW, Gold SE, Müller O, Perlin MH, Wösten HA, de V, R., Ruiz-Herrera J, Reynaga-Pena CG, Snetselaar K, McCann M, Pérez-Martin J, Feldbrügge M, Basse CW, Steinberg G, Ibeas JI, Holloman W, Guzman P, Farman M, Stajich JE, Sentandreu R, González-Prieto JM, Kennell JC, Molina L, Schirawski J, Mendoza-Mendoza A, Greilinger D, Münch K, Rössel N, Scherer M, Vranes M, Ladendorf O, Vincon V, Fuchs U, Sandrock B, Meng S, Ho EC, Cahill MJ, Boyce K, Klose J, Klosterman SJ, Deelstra HJ, Ortiz-Castellanos L, Li W, Sanchez-Alonso P, Schreier PH, Häuser-Hahn I, Vaupel M, Koopmann E, Friedrich G, Voss H, Schlüter T, Margolis J, Platt D, Swimmer C, Gnirke A, Chen F, Vysotskaia V, Mannhaupt G, Güldener U, Münsterkötter M, Haase D, Oesterheld M, Mewes HW, Mauceli EW, DeCaprio D, Wade CM, Butler J, Young S, Jaffe DB, Calvo S, Nusbaum C, Galagan J, Birren BW (2006) Insights from the genome of the biotrophic fungal plant pathogen *Ustilago maydis* . Nature 444:97-101

Kourist R, Bracharz F, Lorenzen J, Kracht ON, Chovatia M, Daum C, Deshpande S, Lipzen A, Nolan M, Ohm RA, Grigoriev IV, Sun S, Heitman J, Brück T, Nowrousian M (2015) Genomics and transcriptomics of the oil-accumulating basidiomycete yeast *Trichosporon oleaginosus* : insights into substrate utilization and alternative evolutionary trajectories of fungal mating systems. mBio 6:e00918-15

Loftus BJ, Fung E, Roncaglia P, Rowley D, Amedeo P, Bruno D, Vamathevan J, Miranda M, Anderson IJ, Fraser JA, Allen JE, Bosdet IE, Brent MR, Chiu R, Doering TL, Donlin MJ, D'Souza CA, Fox DS, Grinberg V, Fu J, Fukushima M, Haas BJ, Huang JC, Janbon G, Jones SJM, Koo HL, Krzywinski MI, Kwon-Chung JK, Lengeler KB, Maiti R, Marra MA, Marra RE, Mathewson CA, Mitchell TG, Pertea M, Riggs FR, Salzberg SL, Schein JE, Shvartsbeyn A, Shin H, Shumway M, Specht CA, Suh BB, Tenney A, Utterback TR, Wickes BL, Wortman JR, Wye NH, Kronstad JW, Lodge JK, Heitman J, Davis RW, Fraser CM, Hyman RW (2005) The genome of the basidiomycetous yeast and human pathogen *Cryptococcus neoformans* . Science 307:1321-1324

Mondo SJ, Dannebaum RO, Kuo RC, Louie KB, Bewick AJ, LaButti K, Haridas S, Kuo A, Salamov A, Ahrendt SR, Lau R, Bowen BP, Lipzen A, Sullivan W, Andreopoulos BB, Clum A, Lindquist E, Daum C, Northen TR, Kunde-Ramamoorthy G, Schmitz RJ, Gryganskyi A, Culley D, Magnuson JK, James TY, O'Malley MA, Stajich JE, Spatafora JW, Visel A, Grigoriev IV (2017) Widespread adenine N6-methylation of active genes in fungi. Nat Genet 49:964-968

Nguyen HDT, Chabot D, Hirooka Y, Roberson RW, Seifert KA (2015) *Basidioascus undulatus* : genome, origins, and sexuality. IMA Fungus 6:215-231

Nizovoy P, Bellora N, Haridas S, Sun H, Daum C, Barry K, Grigoriev IV, Libkind D, Connell LB, Moliné M (2021) Unique genomic traits for cold adaptation in *Naganishia vishniacii* , a polyextremophile yeast isolated from Antarctica. FEMS Yeast Res 21:foaa056

Passer AR, Coelho MA, Billmyre RB, Nowrousian M, Mittelbach M, Yurkov AM, Floyd Averette A, Cuomo CA, Sun S, Heitman J (2019) Genetic and genomic analyses reveal boundaries between species closely related to Cryptococcus pathogens. mBio 10:e00764-19

Rong X, McSpadden Gardener BB (2013) Draft Genome Sequence of *Cryptococcus flavescens* Strain OH182.9_3C, a Biocontrol Agent against Fusarium Head Blight of Wheat. Genome Announc 1:e00762-13

Sharma R, Gassel S, Steiger S, Xia X, Bauer R, Sandmann G, Thines M (2015) The genome of the basal agaricomycete *Xanthophyllomyces dendrorhous* provides insights into the organization of its acetyl-CoA derived pathways and the evolution of Agaricomycotina. BMC Genomics 16:233

Sriswasdi S, Takashima M, Manabe R, Ohkuma M, Sugita T, Iwasaki W (2016) Global deceleration of gene evolution following recent genome hybridizations in fungi. Genome Res 26:1081-1090

Su Y, Jiang X, Wu W, Wang M, Hamid MI, Xiang M, Liu X (2016) Genomic, transcriptomic, and proteomic analysis provide insights into the cold adaptation mechanism of the obligate psychrophilic fungus *Mrakia psychrophila* . G3 (Bethesda) 6:3603-3613

Sun S, Yadav V, Billmyre RB, Cuomo CA, Nowrousian M, Wang L, Souciet JL, Boekhout T, Porcel B, Wincker P, Granek JA, Sanyal K, Heitman J (2017) Fungal genome and mating system transitions facilitated by chromosomal translocations involving intercentromeric recombination. PLoS Biol 15:e2002527

Sun S, Coelho MA, Heitman J, Nowrousian M (2019) Convergent evolution of linked mating-type loci in basidiomycete fungi. PLoS Genet 15:e1008365

Sun J, Xia Y, Ming D (2020) Whole-genome sequencing and bioinformatics analysis of *Apiotrichum mycotoxinivorans* : predicting putative zearalenone-degradation enzymes. Front Microbiol 11:1866

Takashima M, Manabe R, Iwasaki W, Ohyama A, Ohkuma M, Sugita T (2015) Selection of orthologous genes for construction of a highly resolved phylogenetic tree and clarification of the phylogeny of Trichosporonales species. PLOS One 10:e0131217

Takashima M, Sriswasdi S, Manabe RI, Ohkuma M, Sugita T, Iwasaki W (2018) A Trichosporonales genome tree based on 27 haploid and three evolutionary conserved 'natural' hybrid genomes. Yeast 35:99-111

Takashima M, Manabe RI, Ohkuma M (2019) Draft genome sequences of basidiomycetous epiphytic phylloplane yeast type strains *Dioszegia crocea* JCM 2961 and *Dioszegia aurantiaca* JCM 2956. Microbiol Resour Announc 8:e01727-18

Tsuji M, Kudoh S, Hoshino T (2015) Draft genome sequence of cryophilic basidiomycetous yeast *Mrakia blollopis* SK-4, isolated from an algal mat of Naga-ike lake in the Skarvsnes ice-free area, East Antarctica. Genome Announc 3:e01454-14

Vajpeyi S, Chandran K (2016) Draft genome sequence of the oleaginous yeast *Cryptococcus albidus* var. albidus. Genome Announc 4:e00390-16

Yang RY, Li HT, Zhu H, Zhou GP, Wang M, Wang L (2012) Draft genome sequence of CBS 2479, the standard type strain of *Trichosporon asahii* . Eukaryot Cell 11:1415-1416

Yang RY, Li HT, Zhu H, Zhou GP, Wang M, Wang L (2012) Genome sequence of the *Trichosporon asahii* environmental strain CBS 8904. Eukaryot Cell 11:1586-1587

Yong X, Yan Z, Xu L, Zhou J, Wu X, Wu Y, Li Y, Chen Z, Zhou H, Wei P, Jia H (2016) Genome sequence of a microbial lipid producing fungus *Cryptococcus albidus* NT2002. J Biotechnol 223:6-7

Zajc J, Liu Y, Dai W, Yang Z, Hu J, Gostinčar C, Gunde-Cimerman N (2013) Genome and transcriptome sequencing of the halophilic fungus *Wallemia ichthyophaga* : haloadaptations present and absent. BMC Genomics 14:617

Table S2. Analysis of putative telomeric repeats at contig ends of CBS6241.
The search pattern TTAGGGG had to occur consecutively at least three times.
Contigs with putative telomeric repeats at both ends (7 contigs) are labelled in red.
Contigs with putative telomeric repeats at one end (23 contigs) are labelled in blue.

sequence id	sequence length	positions of forward hit regions (start..end)	positions of reverse hit regions (start..end)
CBS6241_contig01	2287425	2287343..2287363;2287365..2287406	
CBS6241_contig02	1507163		2..36
CBS6241_contig03	1267698	1267613..1267682	1..77
CBS6241_contig04	1224624		7..83
CBS6241_contig05	1165378	1165296..1165358	
CBS6241_contig06	1160470		
CBS6241_contig07	1134552	1134524..1134544	3..51
CBS6241_contig08	1028335	1028252..1028307;1028314..1028334	
CBS6241_contig09	1000752		12..53;55..82
CBS6241_contig10	964826		2..57
CBS6241_contig11	943230	943155..943217	
CBS6241_contig12	938801		7..83
CBS6241_contig13	923723	923647..923716	12..74
CBS6241_contig14	907285	907201..907277	7..90
CBS6241_contig15	886904		1..35
CBS6241_contig16	832575	832492..832575	2..71
CBS6241_contig17	763095	762997..763094	5..60
CBS6241_contig18	720935		
CBS6241_contig19	713492		
CBS6241_contig20	700010		12..74
CBS6241_contig21	662110	662017..662100	
CBS6241_contig22	630008		
CBS6241_contig23	623289	623199..623282	
CBS6241_contig24	616656		2..71
CBS6241_contig25	555336		
CBS6241_contig26	554712	554635..554711	
CBS6241_contig27	545542		6..82
CBS6241_contig28	543239		
CBS6241_contig29	414177	414109..414129;414136..414177	
CBS6241_contig30	322761	322679..322755	
CBS6241_contig31	191477		
CBS6241_contig32	184136		1..63
CBS6241_contig33	164611		4..52;59..79
CBS6241_contig34	103872		4..87
CBS6241_contig35	100609	100567..100587	2..50
CBS6241_contig36	68702		2..29
CBS6241_contig37	37757	37692..37747	
CBS6241_contig38	21328		
CBS6241_contig39	15959		
CBS6241_contig40	15573		
CBS6241_contig41	14394		
CBS6241_contig42	11571		

Table S3: Analysis of unique orthogroups in *F. floriforme*

eggNOG category	Total	Orthogroup	PFAM Description	copy number CBS6241	copy number CBS6242
**METABOLISM**	**20**				
Amino acid transport and metabolism [E]	4	OG0016003	Acetyltransferase (GNAT) family	2	1
		OG0013129	Amidinotransferase	2	2
		OG0021765	H-type lectin domain	1	1
		OG0006412	Pyridoxal-dependent decarboxylase conserved domain	1	1
Carbohydrate transport and metabolism [G]	13	OG0010955	Alpha amylase, catalytic domain	1	1
		OG0013121	Alpha-kinase family	2	1
		OG0013149	Alpha-kinase family	2	2
		OG0022209	Glycosyl hydrolases family 31	1	1
		OG0006021	Glycosyl hydrolases family 43	1	1
		OG0021360	Glyoxalase/Bleomycin resistance protein/Dioxygenase superfamily	1	1
		OG0005127	HpcH/HpaI aldolase/citrate lyase family	2	2
		OG0013070	Isochorismatase family	1	1
		OG0005544	Major Facilitator Superfamily	2	2
		OG0006721	Major Facilitator Superfamily	2	3
		OG0007163	Major Facilitator Superfamily	1	1
		OG0007791	Pectate lyase	1	1
		OG0009065	Sulfatase	1	1
Secondary metabolites biosynthesis, transport, and catabolism [Q]	3	OG0022199	Alcohol dehydrogenase GroES-like domain	1	1
		OG0013152	AMP-binding enzyme	1	1
		OG0005766	UDP-glucoronosyl and UDP-glucosyl transferase	1	1
**INFORMATION STORAGE AND PROCESSING**	**11**				
Transcription [K]	4	OG0013114	Acetyltransferase (GNAT) family	2	2
		OG0016003	Acetyltransferase (GNAT) family	2	1
		OG0022204	SAD/SRA domain	1	1
		OG0010990	Helicase conserved C-terminal domain	3	2
Replication, recombination and repair [L]	8	OG0007634	Helicase conserved C-terminal domain	2	7
		OG0005661	6-O-methylguanine DNA methyltransferase, DNA binding domain	1	1
		OG0021038	AAA domain	1	1
		OG0007634	DEAD/DEAH box helicase	2	7
		OG0021121	DNA mismatch repair protein, C-terminal domain	1	1
		OG0013147	RNase H-like domain found in reverse transcriptase	2	2
		OG0016013	SNF2 family N-terminal domain	2	1
**CELLULAR PROCESSES AND SIGNALING**	**21**				
Cell wall/membrane/envelope biogenesis [M]	1	OG0021772	Bacterial transferase hexapeptide (six repeats)	1	1
Cell motility [N]	1	OG0022177	Myosin head (motor domain)	1	1
Post-translational modification, protein turnover, and chaperones [O]	10	OG0006549	Dolichol phosphate-mannose biosynthesis regulatory protein (DPM2)	1	1
		OG0014590	Eukaryotic aspartyl protease	1	1
		OG0022153	Fungal specific transcription factor domain	1	1
		OG0021899	Fungal Zn(2)-Cys(6) binuclear cluster domain	1	1
		OG0015959	Isoprenylcysteine carboxyl methyltransferase (ICMT) family	1	1
		OG0006525	Peptidase family M28	1	1
		OG0022142	Peptidase family M48	1	1
		OG0009879	Ulp1 protease family, C-terminal catalytic domain	2	4
		OG0021088	zinc-RING finger domain	1	1
		OG0021068	Zn-finger in ubiquitin-hydrolases and other protein	1	1
Signal transduction mechanisms [T]	7	OG0021890	Histidine kinase-, DNA gyrase B-, and HSP90-like ATPase	1	1
		OG0008760	Mature-T-Cell Proliferation I type	1	1
		OG0006074	PB1 domain	1	1
		OG0007790	Protein kinase domain	4	4
		OG0021479	Protein kinase domain	1	1
		OG0021537	Protein kinase domain	1	1
		OG0021892	Protein kinase domain	1	1
Cytoskeleton [Z]	2	OG0010996	Myosin head (motor domain)	5	0
		OG0010350	Pleckstrin homology domain	1	1
**POORLY CHARACTERIZED**	**31**				
Function unknown [S]	31	OG0007139	Alpha/beta hydrolase family	1	1
		OG0007639	Alpha/beta hydrolase family	1	1
		OG0021339	Alpha/beta hydrolase family	1	1
		OG0021283	Beta-lactamase superfamily domain	1	1
		OG0021734	Costars	1	1
		OG0005240	DHHC palmitoyltransferase	1	1
		OG0014737	Domain of unknown function (DUF1996)	1	1
		OG0008159	DUF218 domain	1	1
		OG0021284	FAD binding domain	1	1
		OG0021899	Fungal Zn(2)-Cys(6) binuclear cluster domain	1	1
		OG0006048	GDSL-like Lipase/Acylhydrolase	1	1
		OG0010953	GDSL-like Lipase/Acylhydrolase family	1	1
		OG0021920	Glycosyl hydrolase catalytic core	1	1
		OG0006850	MULE transposase domain	8	5
		OG0021417	Myb/SANT-like DNA-binding domain	2	0
		OG0005433	NADH:ubiquinone oxidoreductase, NDUFS5-15kDa	1	1
		OG0022149	Nicotianamine synthase protein	1	1
		OG0022193	NmrA-like family	1	1
		OG0018773	Protein of unknown function (DUF1242)	1	0
		OG0013102	Protein of unknown function (DUF962)	1	1
		OG0021646	Putative esterase	1	1
		OG0006131	Seed maturation protein	1	1
		OG0021544	SnoaL-like domain	1	1
		OG0021915	SnoaL-like domain	1	1
		OG0021684	ssDNA-binding domain of telomere protection protein	1	1
		OG0021979	Stress responsive A/B Barrel Domain	1	1
		OG0006755	Transferase family	1	1
		OG0009397	WD domain, G-beta repeat	1	1
		OG0012348	Winged helix-turn helix	1	0
		OG0008383	YKOF-related Family	1	1
		OG0021278	Zinc finger, ZZ type	1	1

Text S1. Perl script for searching for putative pheromone genes.

The script opens a user-specified fasta file with DNA sequences as input, translates the sequences in all possible reading frames and searches for genes that might encode a lipopeptide pheromone according to the following criteria:

1. starts with a methionin
2. peptide length 20-65 amino acids
3. CAAX motif at the C-terminus before the stop codon, i.e. a protein with the structure M-X(15-60)-CAAX-Stop
   In this case, A stands for the aliphatic amino acids valin (V), leucin (L), isoleucin (I) and methionin (M). In addition, the amino acids threonin (T) and serine (S) are also allowed in these positions since they occur in some putative pheromones of Trichosporonales. X stands for any amino acid.

Please note that this script does not find putative pheromone genes with introns that would result in stop codons when translated (splicing information is not incorporated).

The source code of the perl script is as follows:

```
#!/usr/bin/perl -w

# Copyright (C) 2021 Minou Nowrousian

# This program is free software: you can redistribute it and/or modify
# it under the terms of the GNU General Public License as published by
# the Free Software Foundation, either version 3 of the License, or
# (at your option) any later version.

# This program is distributed in the hope that it will be useful,
# but WITHOUT ANY WARRANTY; without even the implied warranty of
# MERCHANTABILITY or FITNESS FOR A PARTICULAR PURPOSE.  See the
# GNU General Public License for more details (http://www.gnu.org/licenses/).

use strict;
use warnings;

sub datei_einlesen { #reads file into array
   my $quelldatei;
   my @zeilen;
   $quelldatei = shift @_;
   open QUELLE, $quelldatei
      or die "Quelldatei nicht gefunden: $!";
   @zeilen = <QUELLE>;
   close QUELLE;
   return @zeilen;
}

sub fasta_einzeiler_erzeugen {
#generates array with fasta ID as first element and sequence as second

   my $zeile = shift @_;
   $zeile =~ s/\s+$//;  #remove all trailing whitespace, end of line, tabs etc.
   my @einzeiler;
   my $nur_sequenz;
   push (@einzeiler, $zeile);  # first line has to be fasta-identifier
   while (@_) {
      $zeile = shift @_;
      $zeile =~ s/\s+$//;
      if ($zeile =~ />/) {
         push (@einzeiler, $nur_sequenz);
         $nur_sequenz = ();
         push (@einzeiler, $zeile);
      } else {
         $nur_sequenz = $nur_sequenz.$zeile;
      }
   }
```

```
   push (@einzeiler, $nur_sequenz);
   return (@einzeiler);
}


sub translate_cds {
# the subroutine takes two parameters, first the DNA sequence, then the
# information about the type of genome.
# It translates everything according to standard genetic code,
# only if type of genome is "mito",
# then TGA is translated as W instead of stop (fungal mitochondria)

   my $cds = shift(@_);
   my $type = shift(@_);
   my $peptide;

   until (length($cds) < 3) {
      $cds =~ s/(.{3})//;
      my $triplet = $1;

      if ($triplet =~ /GCA|GCC|GCG|GCT/i) {
         $peptide .= "A";
      } elsif ($triplet =~ /TGC|TGT/i) {
         $peptide .= "C";
      } elsif ($triplet =~ /GAC|GAT/i) {
         $peptide .= "D";
      } elsif ($triplet =~ /GAA|GAG/i) {
         $peptide .= "E";
      } elsif ($triplet =~ /TTC|TTT/i) {
         $peptide .= "F";
      } elsif ($triplet =~ /GGA|GGC|GGG|GGT/i) {
         $peptide .= "G";
      } elsif ($triplet =~ /CAC|CAT/i) {
         $peptide .= "H";
      } elsif ($triplet =~ /ATA|ATC|ATT/i) {
         $peptide .= "I";
      } elsif ($triplet =~ /AAA|AAG/i) {
         $peptide .= "K";
      } elsif ($triplet =~ /CTA|CTC|CTG|CTT|TTA|TTG/i) {
         $peptide .= "L";
      } elsif ($triplet =~ /ATG/i) {
         $peptide .= "M";
      } elsif ($triplet =~ /AAC|AAT/i) {
         $peptide .= "N";
      } elsif ($triplet =~ /CCA|CCC|CCG|CCT/i) {
         $peptide .= "P";
      } elsif ($triplet =~ /CAA|CAG/i) {
         $peptide .= "Q";
      } elsif ($triplet =~ /AGA|AGG|CGA|CGC|CGG|CGT/i) {
         $peptide .= "R";
      } elsif ($triplet =~ /AGC|AGT|TCA|TCC|TCG|TCT/i) {
         $peptide .= "S";
      } elsif ($triplet =~ /ACA|ACC|ACG|ACT/i) {
         $peptide .= "T";
      } elsif ($triplet =~ /GTA|GTC|GTG|GTT/i) {
         $peptide .= "V";
      } elsif ($triplet =~ /TGG/i) {
         $peptide .= "W";
      } elsif ($triplet =~ /TAC|TAT/i) {
         $peptide .= "Y";
      } elsif ($triplet =~ /TAA|TAG/i) {
         $peptide .= "*";
      } elsif ($triplet =~ /TGA/i) {
         if ($type =~ /mito/) {
            $peptide .= "W";
         } else {
            $peptide .= "*";
         }
      } else {
         $peptide .= "X";
```

```
      }
   }
   return($peptide);
}

sub reverse_complement { #makes reverse complement of sequence string
   my $seq = shift @_;
   my $rev = reverse($seq);
   $rev =~ tr/ACGTacgt/TGCAtgca/; ## translate operator
   return $rev;
}

print "The input file has to be in folder seqs.\n\n";
print "Please give name of input fasta file:\n";
chomp (my $file = <STDIN>);

print "Please give name for output table file with putative pheromones:\n";
chomp (my $outtab = <STDIN>);

print "Please give name for output fasta file with putative pheromones:\n";
chomp (my $outfasta = <STDIN>);

chdir "seqs" or die "chdir nach seqs nicht moeglich: $!";

open OUTTAB, ">> $outtab" or die "cannot open file: $!";
print OUTTAB "contig\tstrand\tstart\tend\tdna_length\tput_pheromone\n";

open OUTFASTA, ">> $outfasta" or die "Kann Datei nicht oeffnen: $!";

my @datei = &datei_einlesen($file);
my @fasta_einzeiler = &fasta_einzeiler_erzeugen(@datei);

## make hash with fasta-id as key and seq as value
my %fasta = @fasta_einzeiler;

# now translate each contig in all six frames and search each
# frame for the consensus sequence

foreach my $contig (sort keys %fasta) {

   print "starting with contig $contig\n";

   my $seq = $fasta{$contig};
   my $contiglength = length($seq);
   my $revseq = &reverse_complement($seq);
   my @frames = ($seq, $revseq);

   $seq = substr($seq, 1);
   # take everything starting at second nucleotide
   $revseq = substr($revseq, 1);
   push(@frames, $seq, $revseq);

   $seq = substr($seq, 1);
   # take everything starting at third nucleotide
   # (counting from original sequence)
   $revseq = substr($revseq, 1);
   push(@frames, $seq, $revseq);

   my $number = 0;

   foreach my $dna (@frames) {

      $number++;
      ## counts the frames for later calculation of position on DNA
      ## the six frames are in the order 1, -1, 2, -2, 3, -3

      my $peptide = &translate_cds($dna, "nucl");
      ## DNA is nuclear, not mitochondrial,
      ## therefore use standard genetic code in subroutine
```

```
      # search for consensus sequence M-X(15-60)-CAAX-Stop

      while ($peptide =~ /M[A-Z]{15,60}C[ILMVST][ILMVST][A-Z]\*/g) {

         my $put_pheromone = $&;
         my $dna_length = length($put_pheromone) * 3;
         ## length of coding sequence for putative pheromone
         my $length_before = length($`) * 3;
         ## length of dna sequence upstream of the consensus
         ## that was found
         my $strand;

         print "found putative pheromone $put_pheromone on $contig\n";

         my ($start, $end);

         if ($number > 4) { ## frame is 3 or -3

            if ($number == 5) {  ## frame is 3
               $start = $length_before + 3;
               $end = $start + $dna_length - 1;
               $strand = "forward";
            } else { ## frame is -3
               $end = $contiglength - $length_before - 2;
               $start = $end - $dna_length + 1;
               $strand = "reverse";
            }

         } elsif ($number > 2) {  ## frame is 2 or -2

            if ($number == 3) {  ## frame is 2
               $start = $length_before + 2;
               $end = $start + $dna_length - 1;
               $strand = "forward";
            } else { ## frame is -2
               $end = $contiglength - $length_before - 1;
               $start = $end - $dna_length + 1;
               $strand = "reverse";
            }

         } else {  ## frame is 1 or -1

            if ($number == 1) { ## frame is 1
               $start = $length_before + 1;
               $end = $start + $dna_length - 1;
               $strand = "forward";
            } else { ## frame is -1
               $end = $contiglength - $length_before;
               $start = $end - $dna_length + 1;
               $strand = "reverse";
            }
         }

         my $t = "\t";
         my $line = $contig.$t.$strand.$t.$start.$t.$end.$t;
         my $line .= $dna_length.$t.$put_pheromone."\n";

         print OUTTAB $line;
         if ($strand eq "forward") {
            $line = "$contig $start..$end\n$put_pheromone\n";
         } else {
            $line = "$contig complement($start..$end)\n$put_pheromone\n";
         }
         print OUTFASTA $line;
      }
   }
}

close OUTTAB;
close OUTFASTA;
```

Text S2. Perl script for searching for putative telomeric repeats.

The script opens a user-specified fasta file with DNA sequences and a user-defined sequence to search for (the expected telomeric repeat sequence) as input and identifies all instances where this sequence occurs at least three times in direct repetition in the input fasta file (in forward and reverse strand). Output is a table with contig lengths and occurrences (contig, position, strand) of the putative telomeric repeat sequence. Since the program finds an instances of three consecutive repetitions of the search sequence, it is up to the user to decide (based on the positions of the identified repeat occurrences within the contigs) if the repeat occurrence might represent true telomeric repeats.

The source code of the perl script is as follows:

```
#!/usr/bin/perl -w

# Copyright (C) 2021 Minou Nowrousian

# This program is free software: you can redistribute it and/or modify
# it under the terms of the GNU General Public License as published by
# the Free Software Foundation, either version 3 of the License, or
# (at your option) any later version.

# This program is distributed in the hope that it will be useful,
# but WITHOUT ANY WARRANTY; without even the implied warranty of
# MERCHANTABILITY or FITNESS FOR A PARTICULAR PURPOSE.  See the
# GNU General Public License for more details (http://www.gnu.org/licenses/).

use strict;
use warnings;

unless (@ARGV == 2) {
   die("program usage: find_telomeric_repeats.pl inputfile.fasta
   telomeric_repeat_sequence\ninput file has to be in folder seqs");
}

chdir "seqs" or die "chdir nach seqs nicht moeglich: $!";

unless (-e $ARGV[0]) {
   die("Can't open file $ARGV[0]: $!");
}

if ($ARGV[1] =~ /[^acgtACGT]/) {
   die("$ARGV[1] does not look like a DNA sequence!\n");
}


sub datei_einlesen { #reads file into array
   my $quelldatei;
   my @zeilen;
   $quelldatei = shift @_;
   open QUELLE, $quelldatei
      or die "Quelldatei nicht gefunden: $!";
   @zeilen = <QUELLE>;
   close QUELLE;
   return @zeilen;
}

sub fasta_einzeiler_erzeugen {
#generates array with fasta ID as first element and sequence as second

   my $zeile = shift @_;
   $zeile =~ s/\s+$//;  #remove all trailing whitespace, end of line, tabs etc.
   my @einzeiler;
   my $nur_sequenz;
   push (@einzeiler, $zeile);  # first line has to be fasta-identifier
   while (@_) {
      $zeile = shift @_;
      $zeile =~ s/\s+$//;
```

```
      if ($zeile =~ />/) {
         push (@einzeiler, $nur_sequenz);
         $nur_sequenz = ();
         push (@einzeiler, $zeile);
      } else {
         $nur_sequenz = $nur_sequenz.$zeile;
      }
   }
   push (@einzeiler, $nur_sequenz);
   return (@einzeiler);
}

sub reverse_complement { #makes reverse complement of sequence string
   my $seq = shift @_;
   my $rev = reverse($seq);
   $rev =~ tr/ACGTacgt/TGCAtgca/; ## translate operator
   return $rev;
}


my $for_pattern = $ARGV[1];
$for_pattern =~ tr/acgt/ACGT/;
my $rev_pattern = &reverse_complement($for_pattern);

my @fasta = &datei_einlesen($ARGV[0]);
@fasta = &fasta_einzeiler_erzeugen(@fasta);

my %hits_forward = ();
## reference to an anonymous array, key is contig, values are
## length of contig (first value) and then start positions of
## found pattners (putative telomeric repeats) in forward direction,
## add information e.g. with push(@{$hits_forward{$id}}, 10);

my %hits_reverse = ();  ## same for patterns in reverse direction

while (@fasta) {

   my $id = shift(@fasta);
   $id =~ s/\s+$//;     # remove all trailing whitespace, end of line, tabs etc.
   my $seq = shift(@fasta);
   $seq =~ s/\s+$//;
   $seq =~ tr/acgt/ACGT/;

   my $laenge = length($seq);
   push(@{$hits_forward{$id}}, $laenge);

   while ($seq =~ /($for_pattern){3,}/g) {
      my $pos1 = length($`) + 1;
      # the starting position of the match is the length of the section
      # before the match + 1
      my $pos2 = $laenge - length($');
      # the end position of the match is the total length of the sequence
      # minus the lenght of the section after the match
      push(@{$hits_forward{$id}}, "$pos1..$pos2");
   }

   while ($seq =~ /($rev_pattern){3,}/g) {
      my $pos1 = length($`) + 1;
      # the starting position of the match is the length of the section
      # before the match + 1
      my $pos2 = $laenge - length($');
      # the end position of the match is the total length of the
      # sequence minus the lenght of the section after the match
      push(@{$hits_reverse{$id}}, "$pos1..$pos2");
   }

}

my @output = "search file: $ARGV[0]\nsearch pattern: $for_pattern\nsearch pattern
has to occur consecutively at least three times\n\nsequence id\tsequence
```

```
length\tpositions of forward hit regions (start..end)\tpositions of reverse hit
regions (start..end)\n";

for my $id (sort keys %hits_forward) {

   my @values_for;
   my @values_rev;

   if (exists $hits_forward{$id}) {
      @values_for = @{$hits_forward{$id}};
   }

   if (exists $hits_reverse{$id}) {
      @values_rev = @{$hits_reverse{$id}};
   }

   my $laenge = shift(@values_for);
   my $line = "$id\t$laenge\t";

   my $n = @values_for; ## check number of elements
   if ($n > 0) {
      my $values_for = join ";", @values_for;
      $line .= "$values_for\t";
   } else {
      $line .= "\t";
   }


   $n = @values_rev; ## check number of elements
   if ($n > 0) {
      my $values_rev = join ";", @values_rev;
      $line .= "$values_rev\n";
   } else {
      $line .= "\n";
   }

   push(@output, $line);

}

my $filename = $ARGV[0];

if ($filename =~ /\.fasta$/) {
   $filename =~ s/\.fasta$//;
}

$filename .= "_put_telomeric_reps.txt";

open NEU, "> $filename" or die "cannot open file: $!";
print NEU @output;
close NEU;
```
